# Supplementary material for: Laminin-defined mechanical status modulates retinal pigment epithelium phagocytosis
Source: EMBO Rep. 2025 May 19;26(13):3357–83. doi: 10.1038/s44319-025-00475-9 (PMC12238246; doi:10.1038/s44319-025-00475-9)
Supplement: Supplementary file 1 — Table EV1 [file 44319_2025_475_MOESM1_ESM.pdf]

Table EV1

	Laminin 322			Laminin 511		
	Exp 1	Exp 2	Exp 3	Exp 1	Exp 2	Exp 3
5 µg/mL	440 (454)	638 (654)	715 (736)	606 (625)	686 (710)	727 (753)
10 µg/mL	479 (495)	697 (719)	654 (679)	547 (561)	575 (594)	396 (415)
20 µg/mL	627 (641)	683 (698)	608 (634)	559 (572)	831 (856)	480 (499)
30 µg/mL	529 (539)	325 (334)	637 (660)	513 (525)	710 (737)	759 (782)

Table EV1: Number of cells *in vitro* analyzed for morphometric and topological parameters.
