# Supplementary figures and images for: Laminin-defined mechanical status modulates retinal pigment epithelium phagocytosis
Source: EMBO Rep. 2025 May 19;26(13):3357–83. doi: 10.1038/s44319-025-00475-9 (PMC12238246; doi:10.1038/s44319-025-00475-9)

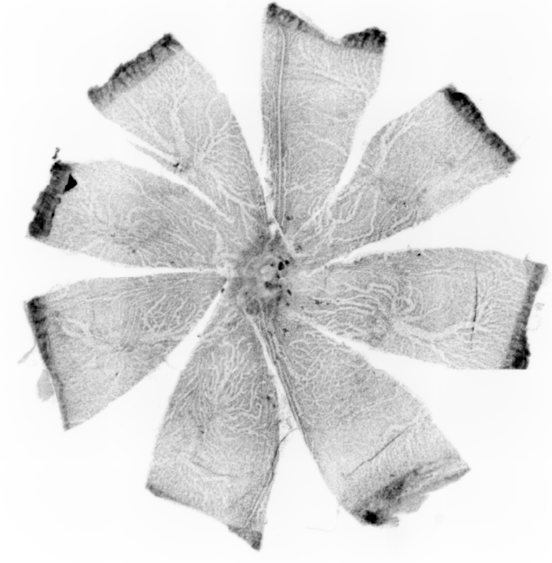

Supplement: Supplementary file 3 — Source data Fig. 1 [file 44319_2025_475_MOESM3_ESM.zip › Figure1/1A/Retina_FlatMount.tif]

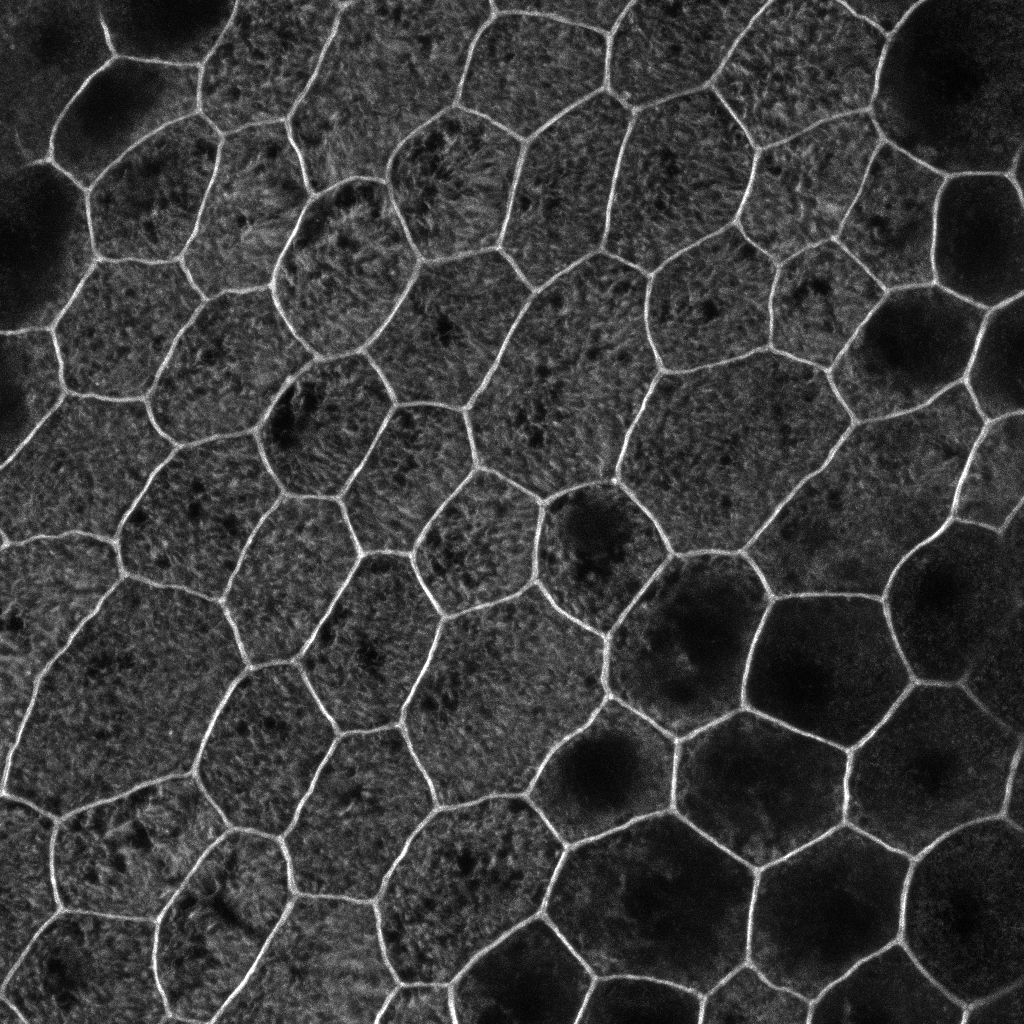

Supplement: Supplementary file 3 — Source data Fig. 1 [file 44319_2025_475_MOESM3_ESM.zip › Figure1/1A/MAX_midPeriphery_image2.tif]

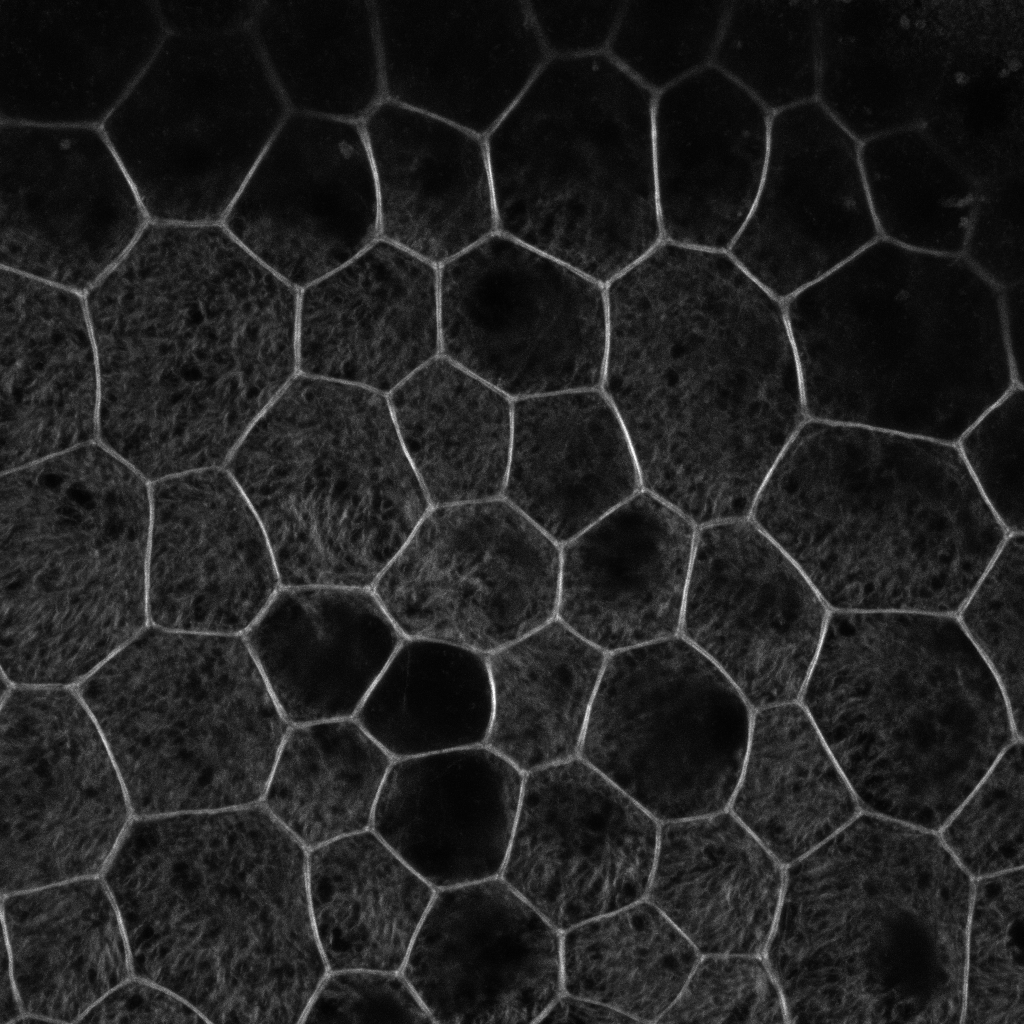

Supplement: Supplementary file 3 — Source data Fig. 1 [file 44319_2025_475_MOESM3_ESM.zip › Figure1/1A/MAX_midPeriphery_image1.tif]

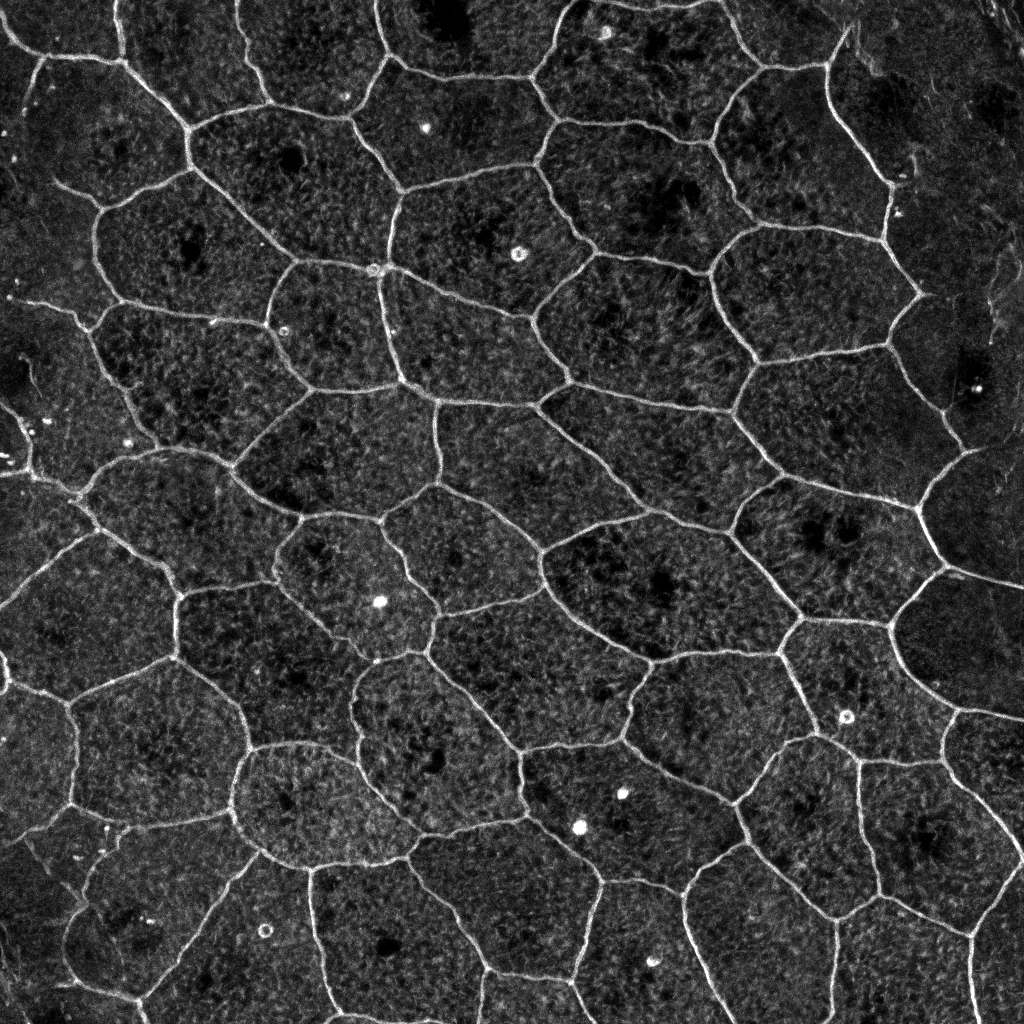

Supplement: Supplementary file 3 — Source data Fig. 1 [file 44319_2025_475_MOESM3_ESM.zip › Figure1/1A/MAX_periphery_image1.tif]

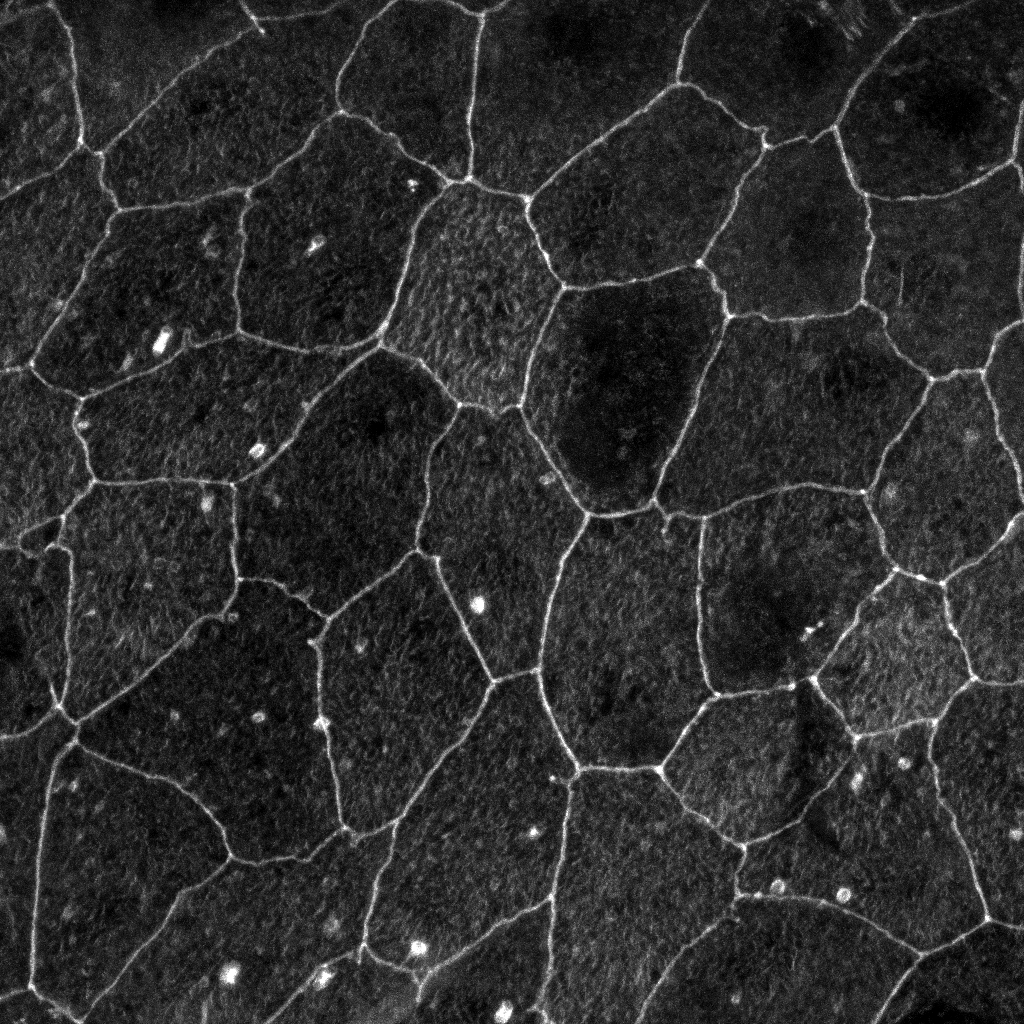

Supplement: Supplementary file 3 — Source data Fig. 1 [file 44319_2025_475_MOESM3_ESM.zip › Figure1/1A/MAX_periphery_image2.tif]

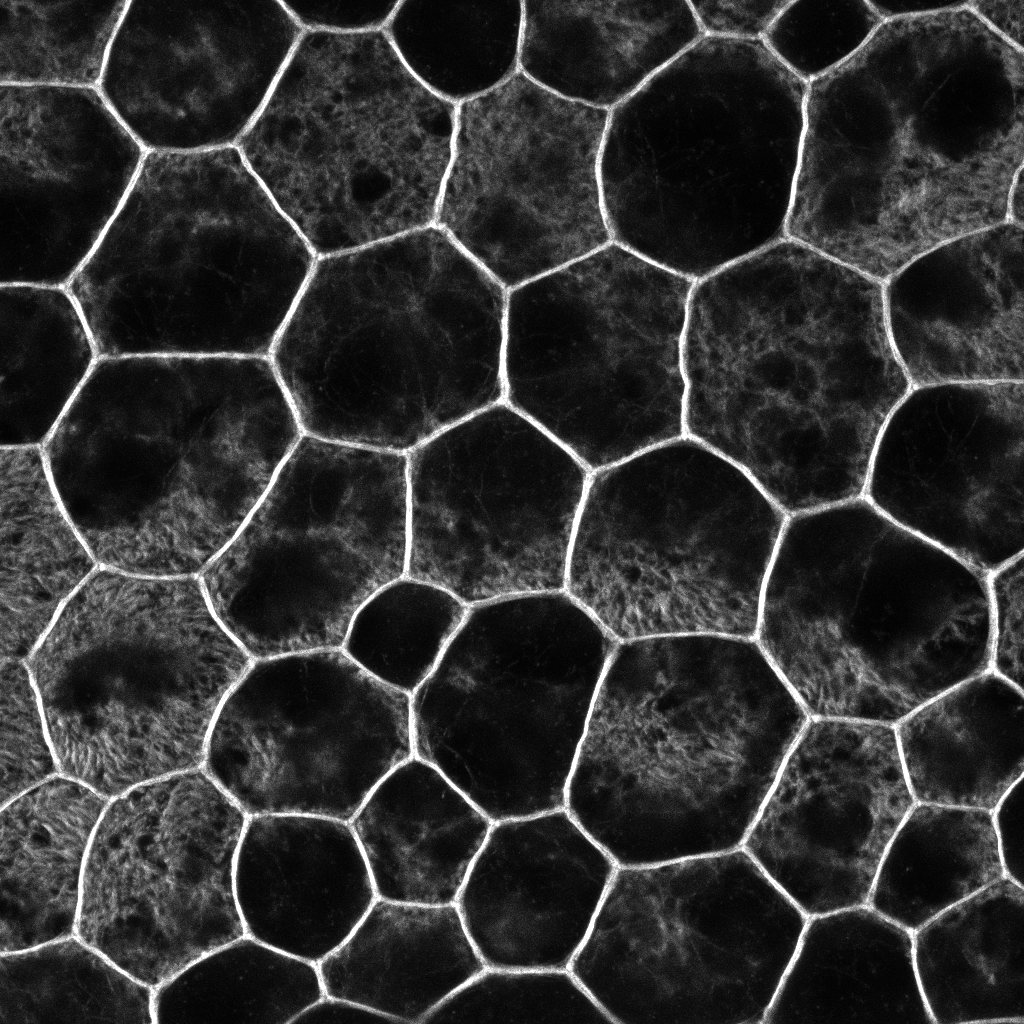

Supplement: Supplementary file 3 — Source data Fig. 1 [file 44319_2025_475_MOESM3_ESM.zip › Figure1/1A/MAX_centre_image2.tif]

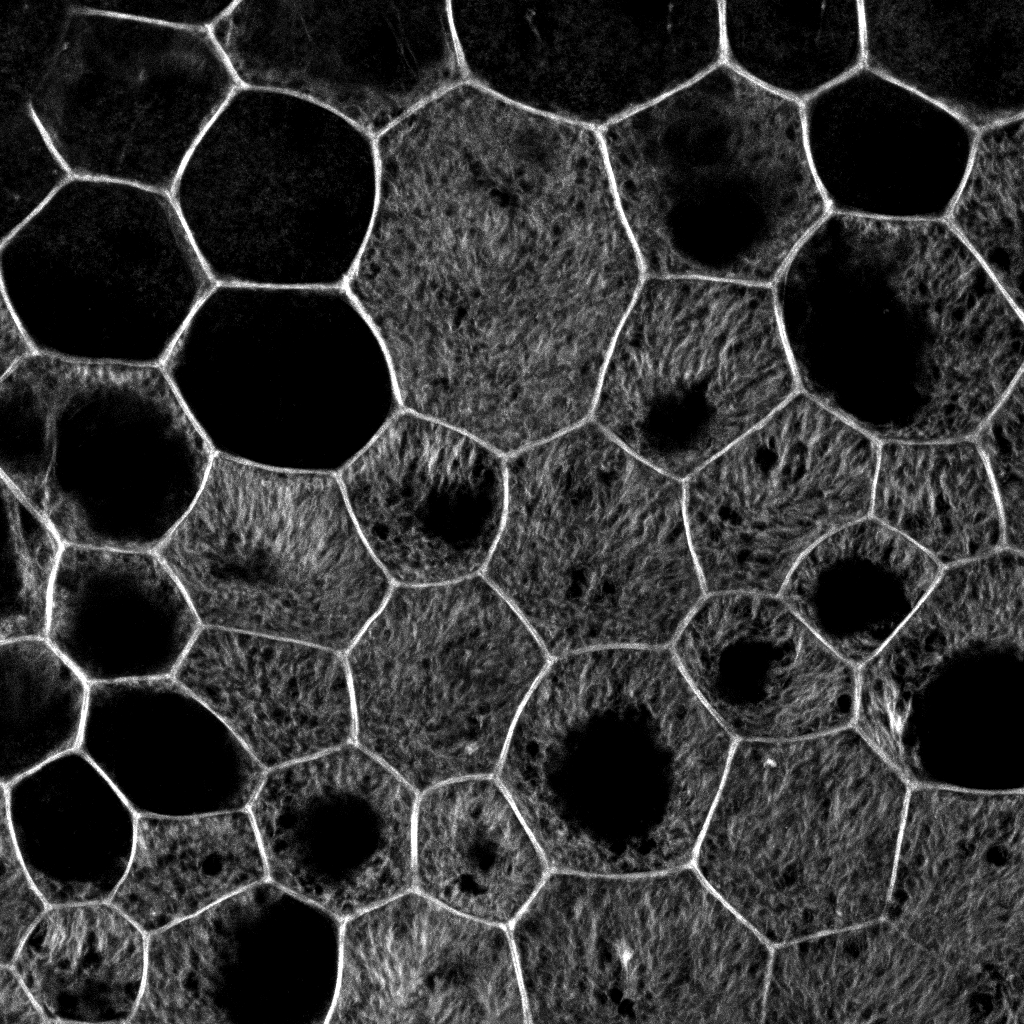

Supplement: Supplementary file 3 — Source data Fig. 1 [file 44319_2025_475_MOESM3_ESM.zip › Figure1/1A/MAX_centre_image1.tif]

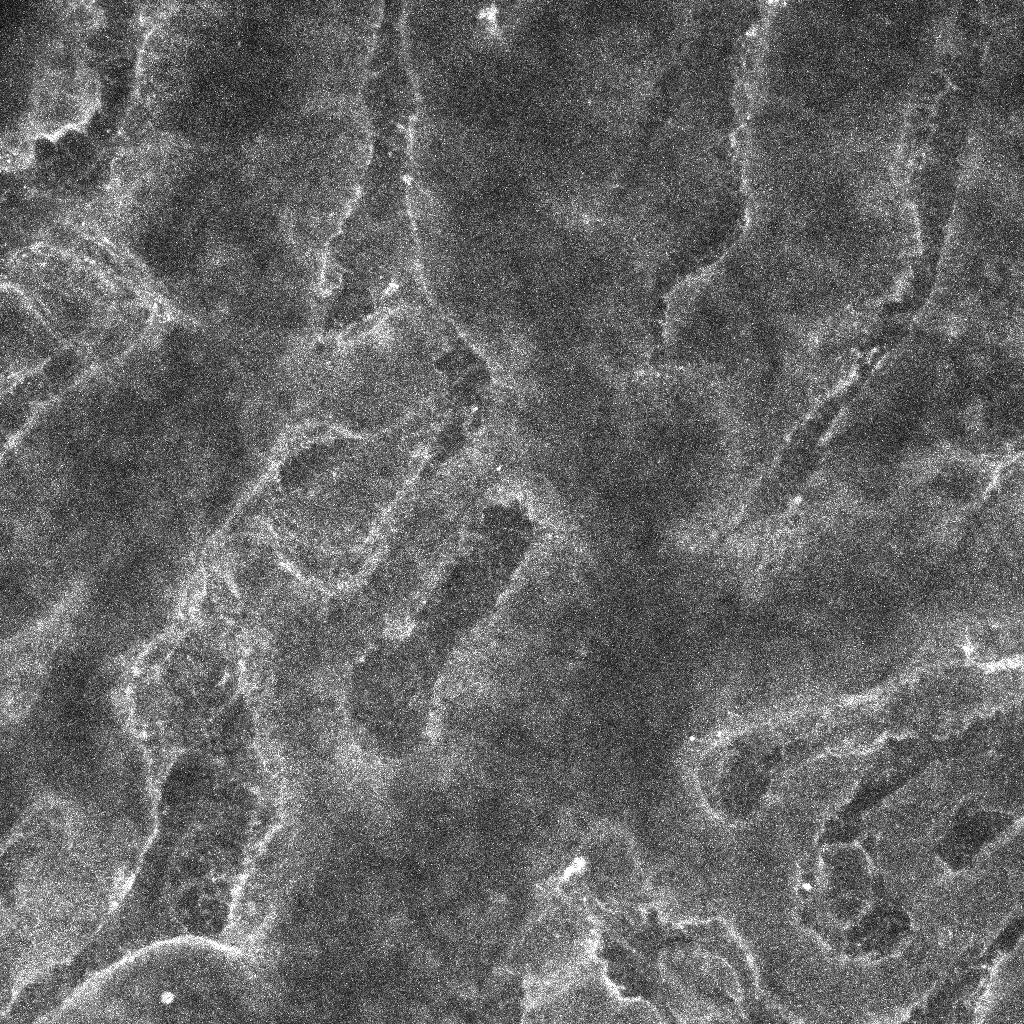

Supplement: Supplementary file 3 — Source data Fig. 1 [file 44319_2025_475_MOESM3_ESM.zip › Figure1/1I/CollagenIV_MAX_midperiphery.tif]

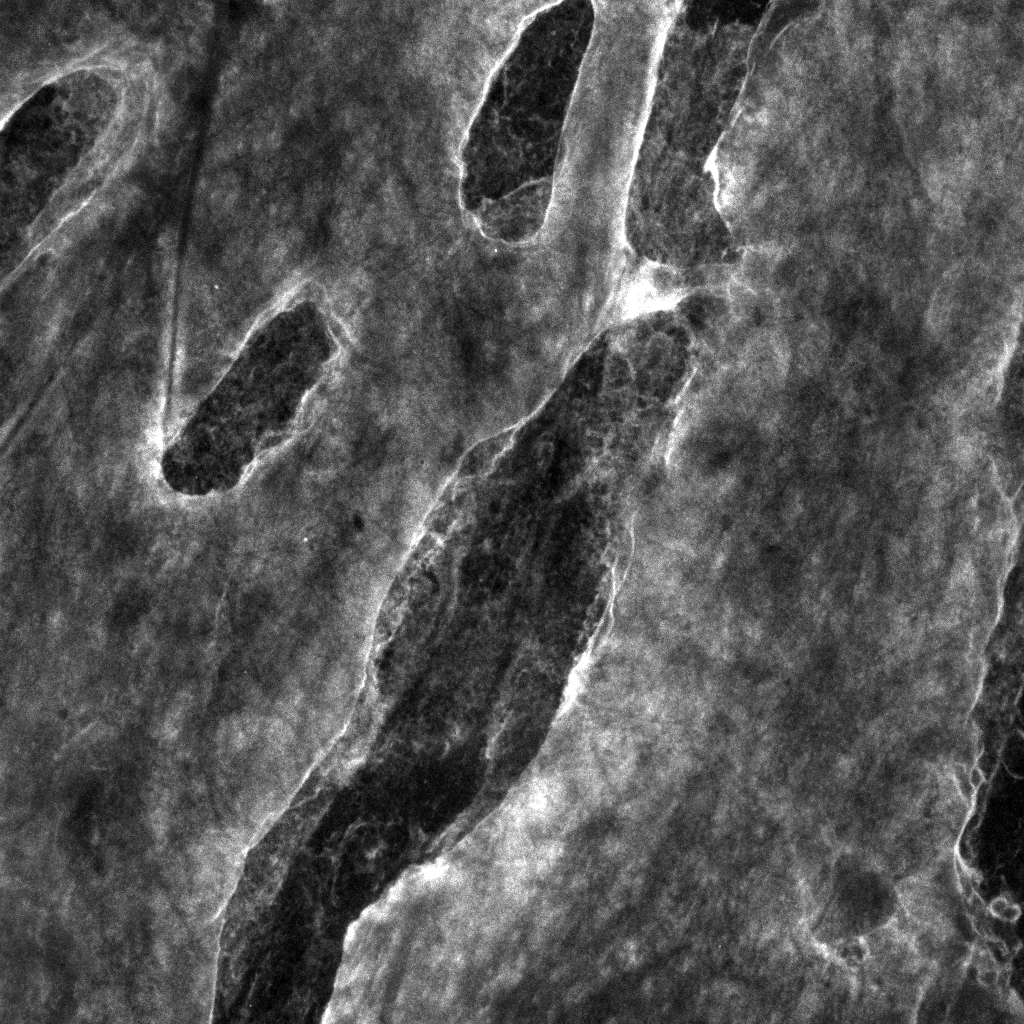

Supplement: Supplementary file 3 — Source data Fig. 1 [file 44319_2025_475_MOESM3_ESM.zip › Figure1/1I/Laminin alpha5_MAX_farperiphery.tif]

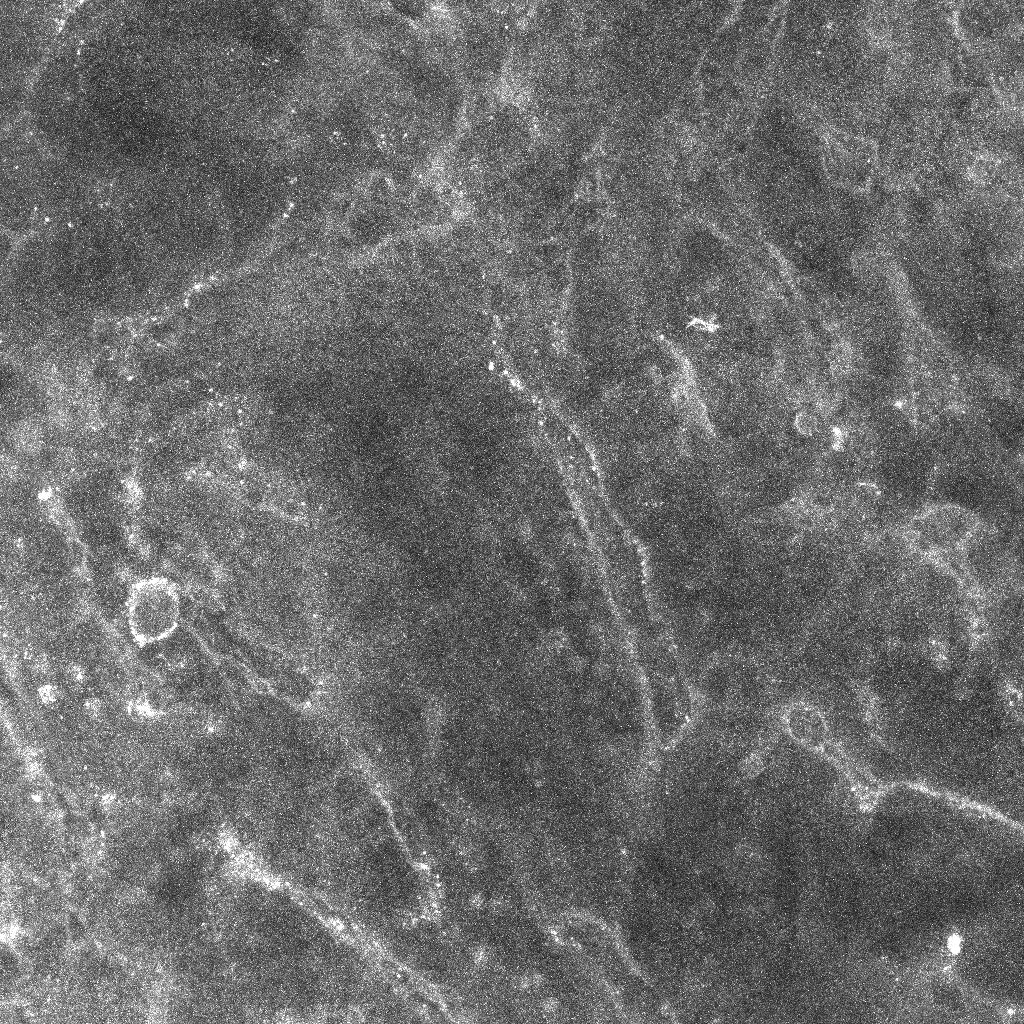

Supplement: Supplementary file 3 — Source data Fig. 1 [file 44319_2025_475_MOESM3_ESM.zip › Figure1/1I/CollagenIV_MAX_centre.tif]

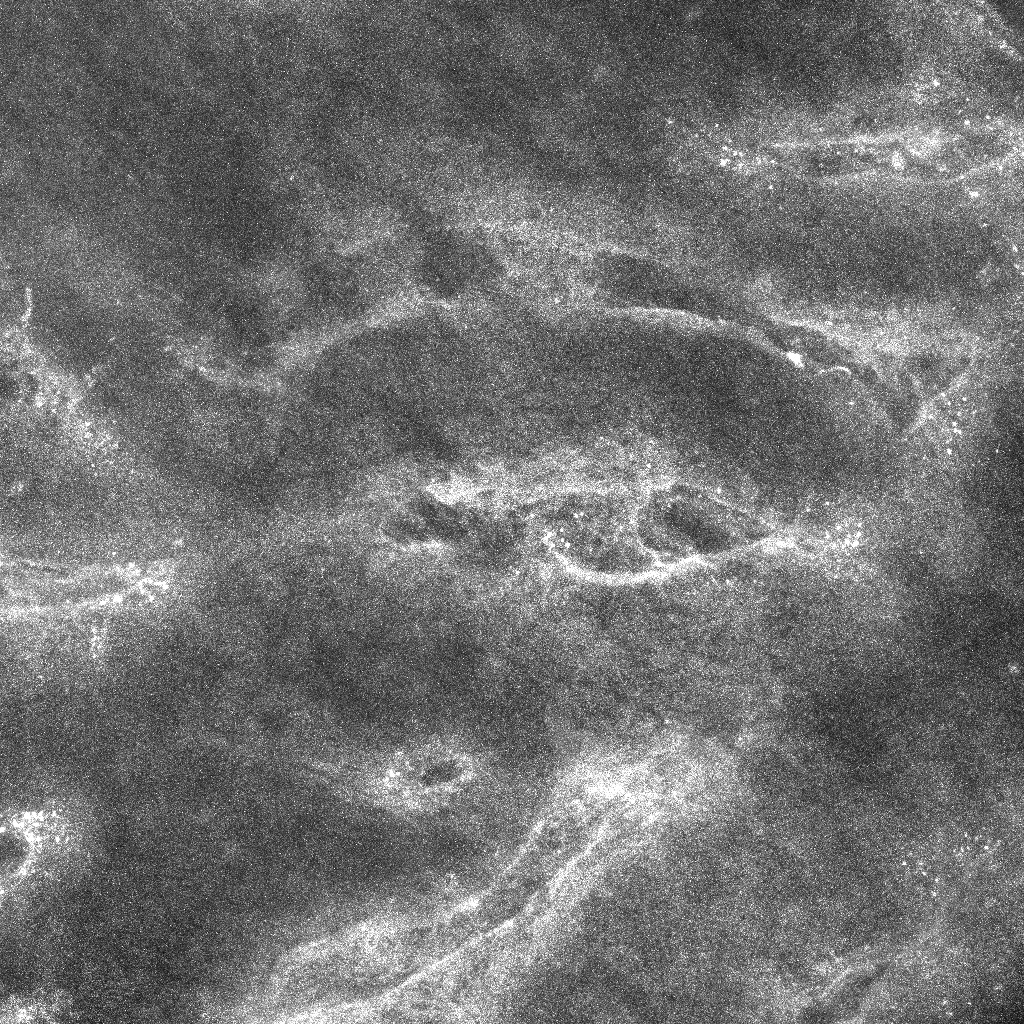

Supplement: Supplementary file 3 — Source data Fig. 1 [file 44319_2025_475_MOESM3_ESM.zip › Figure1/1I/CollagenIV_MAX_farperiphery.tif]

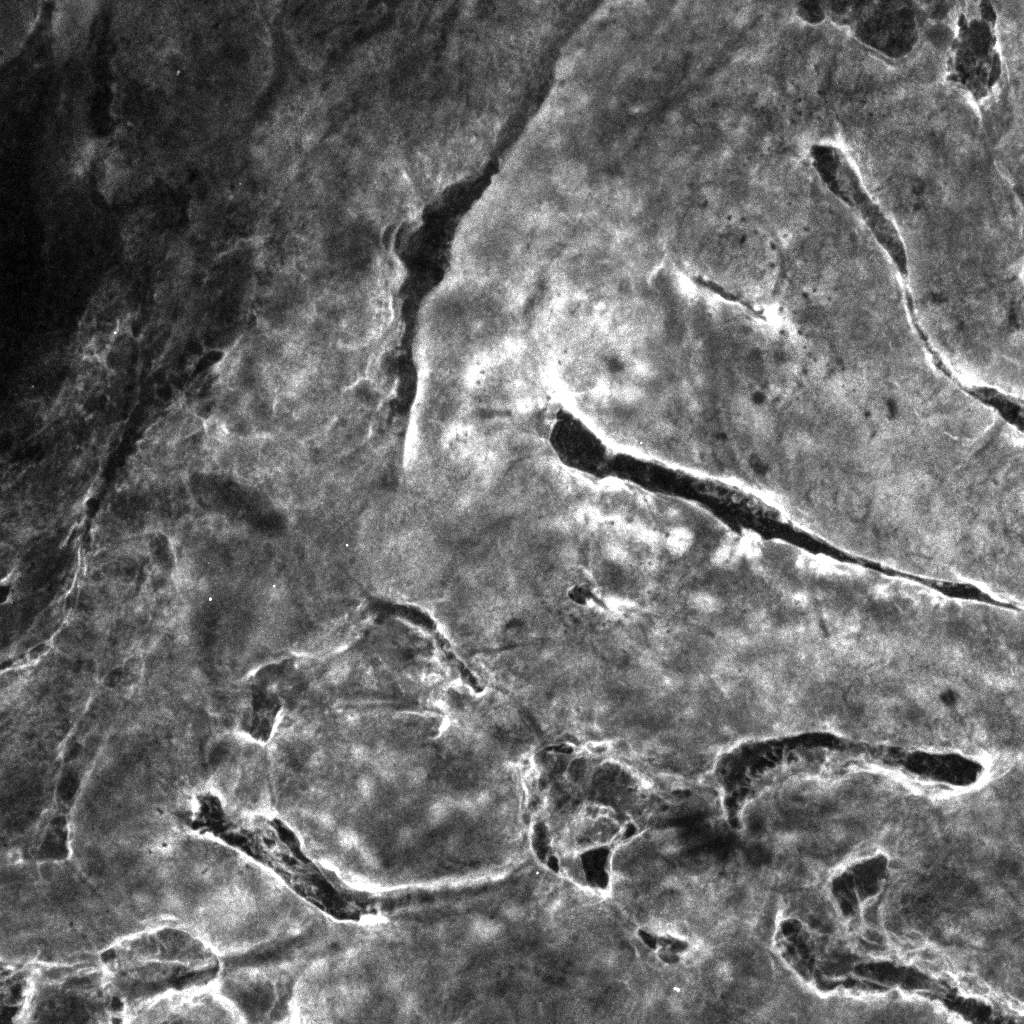

Supplement: Supplementary file 3 — Source data Fig. 1 [file 44319_2025_475_MOESM3_ESM.zip › Figure1/1I/Laminin alpha5_MAX_midperiphery.tif]

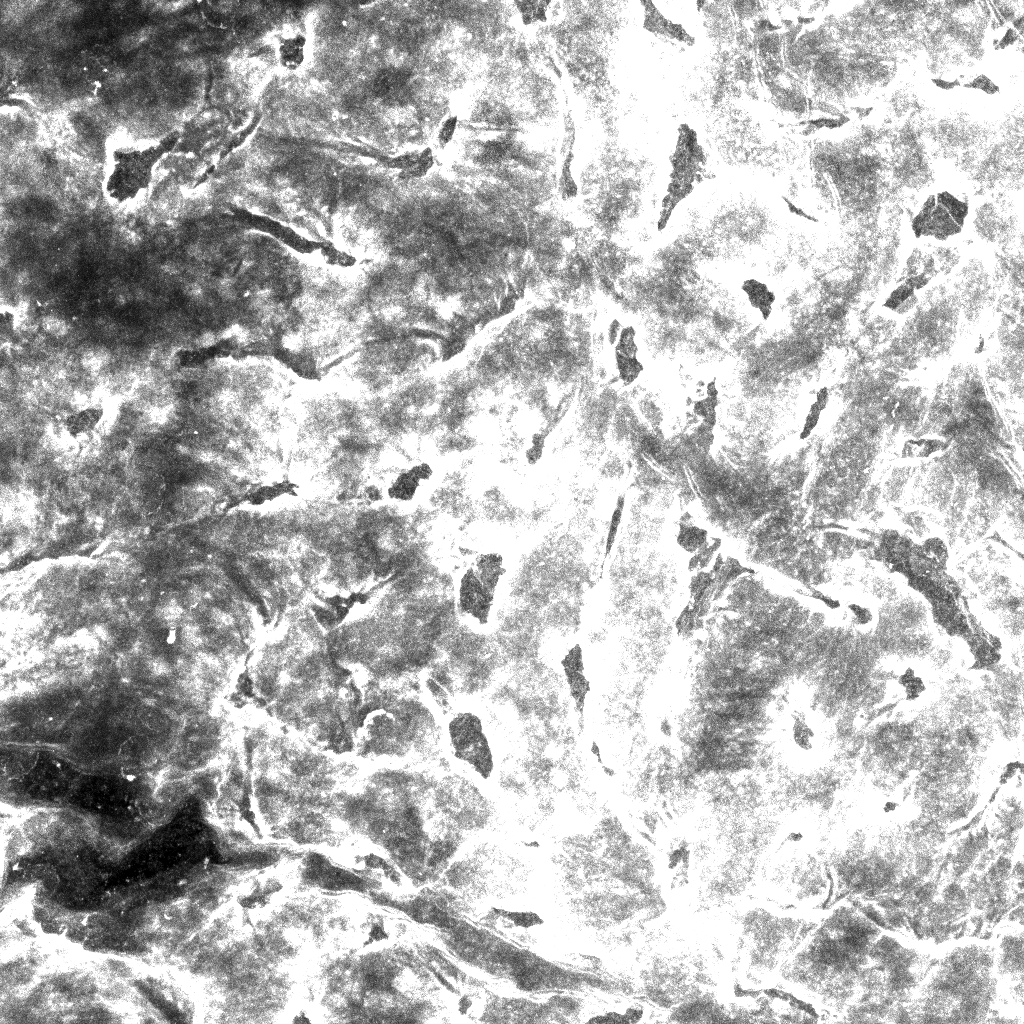

Supplement: Supplementary file 3 — Source data Fig. 1 [file 44319_2025_475_MOESM3_ESM.zip › Figure1/1I/Laminin332_MAX_centre.tif]

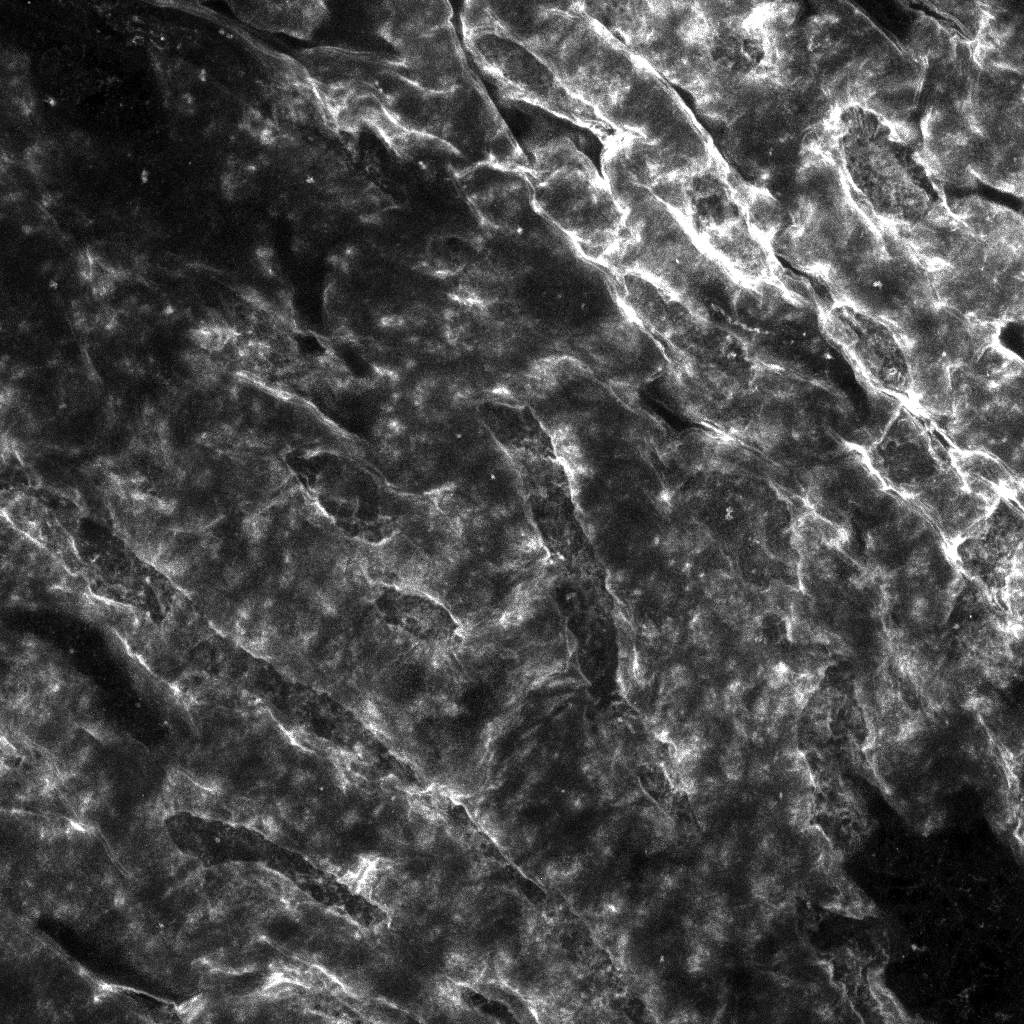

Supplement: Supplementary file 3 — Source data Fig. 1 [file 44319_2025_475_MOESM3_ESM.zip › Figure1/1I/Laminin332_MAX_farperiphery.tif]

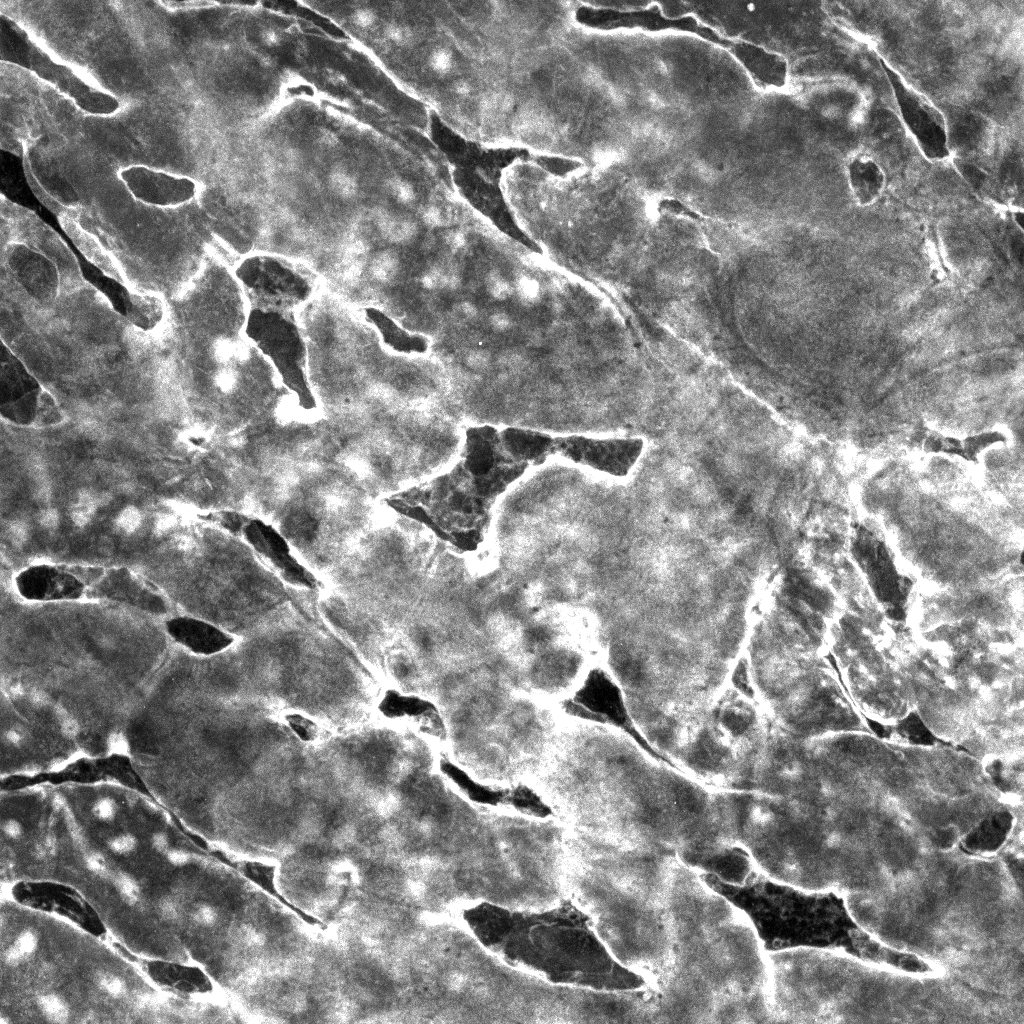

Supplement: Supplementary file 3 — Source data Fig. 1 [file 44319_2025_475_MOESM3_ESM.zip › Figure1/1I/Laminin alpha5_MAX_centre.tif]

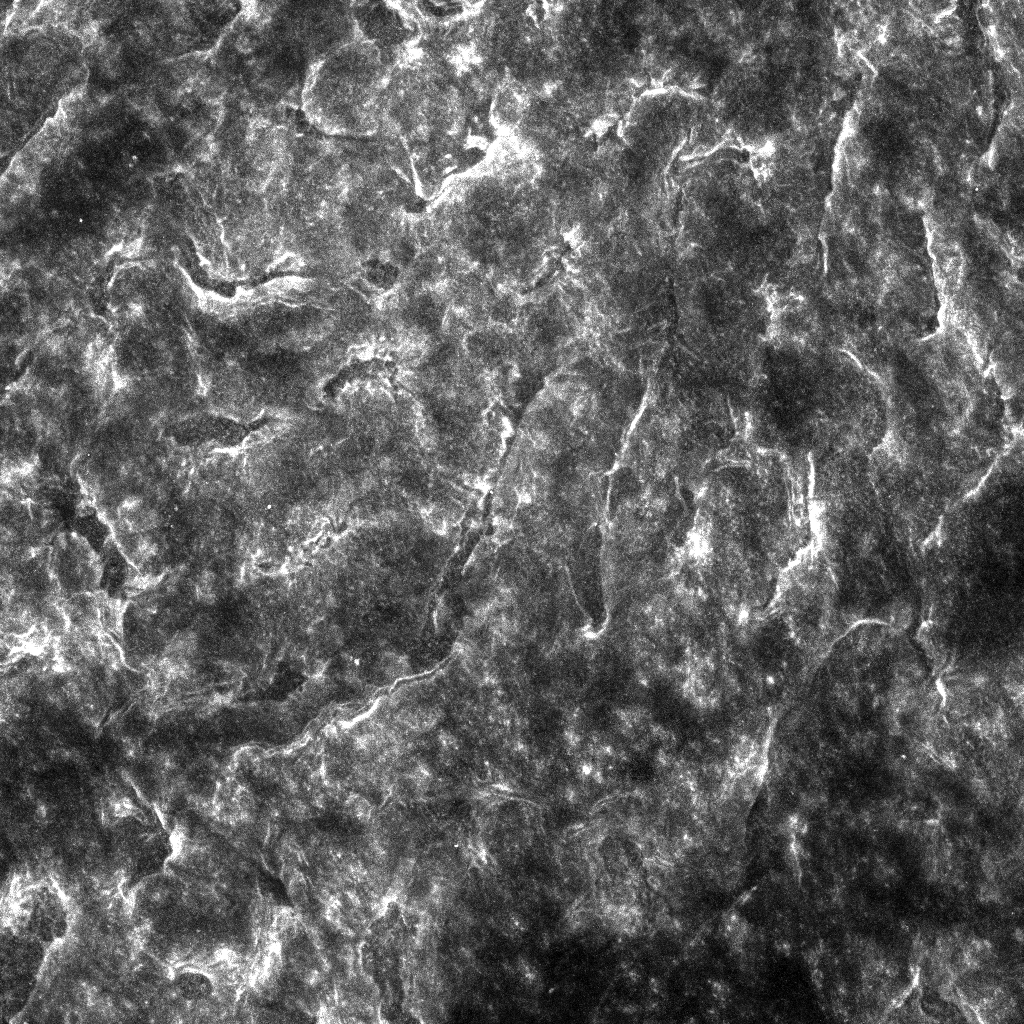

Supplement: Supplementary file 3 — Source data Fig. 1 [file 44319_2025_475_MOESM3_ESM.zip › Figure1/1I/Laminin332_MAX_midperiphery.tif]

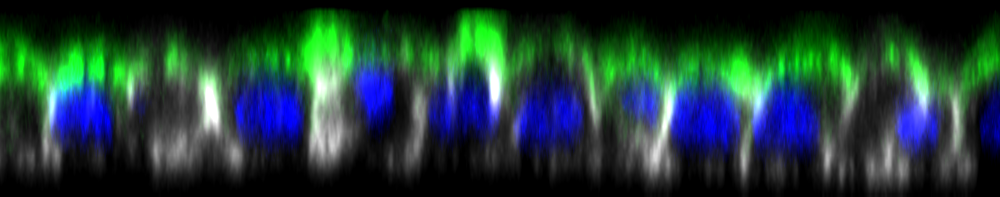

Supplement: Supplementary file 4 — Source data Fig. 2 [file 44319_2025_475_MOESM4_ESM.zip › Figure2/2A/Orthogonal_ Dapi_Ezrin_F-Actin_slice 156.tif]

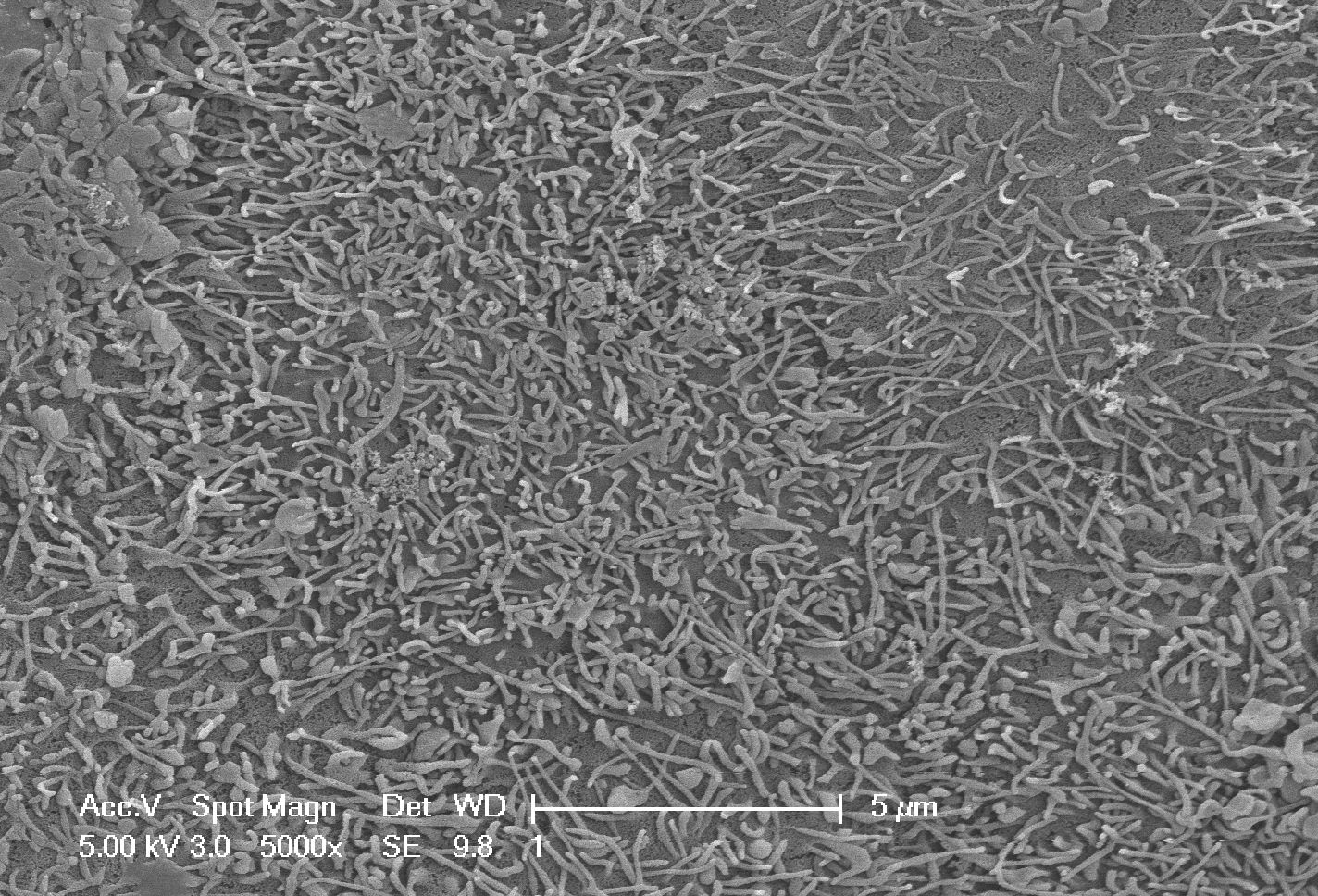

Supplement: Supplementary file 4 — Source data Fig. 2 [file 44319_2025_475_MOESM4_ESM.zip › Figure2/2C/SEM_iRPE.TIF]

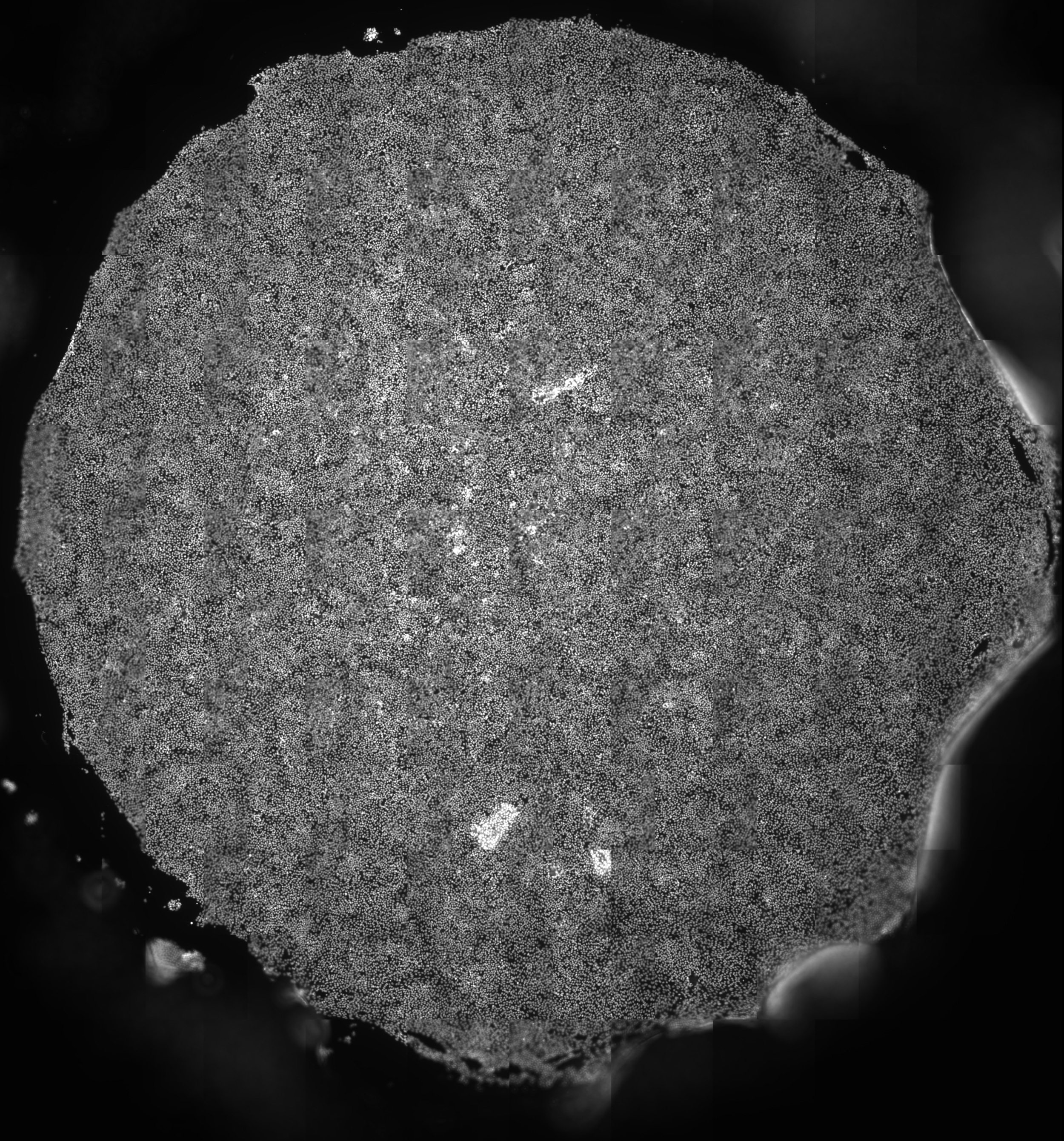

Supplement: Supplementary file 4 — Source data Fig. 2 [file 44319_2025_475_MOESM4_ESM.zip › Figure2/2D/iRPE_Proliferation_Dapi_Overview.tif]

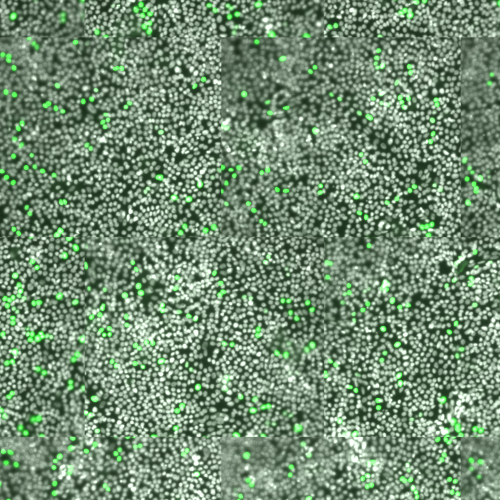

Supplement: Supplementary file 4 — Source data Fig. 2 [file 44319_2025_475_MOESM4_ESM.zip › Figure2/2D/iRPE_Proliferation_Dapi+EdU_Composite.tif]

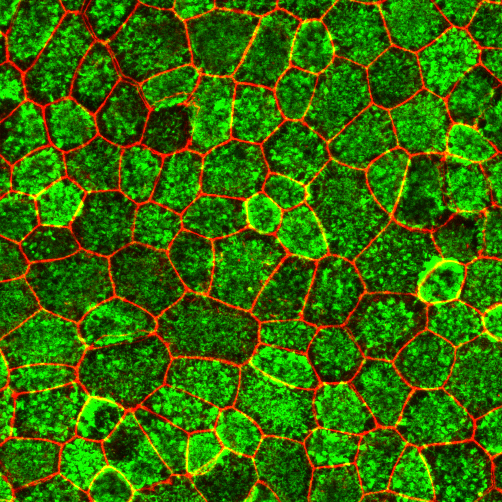

Supplement: Supplementary file 4 — Source data Fig. 2 [file 44319_2025_475_MOESM4_ESM.zip › Figure2/2B/iRPE_Ezrin+ZO-1_ROI_RGB.tif]

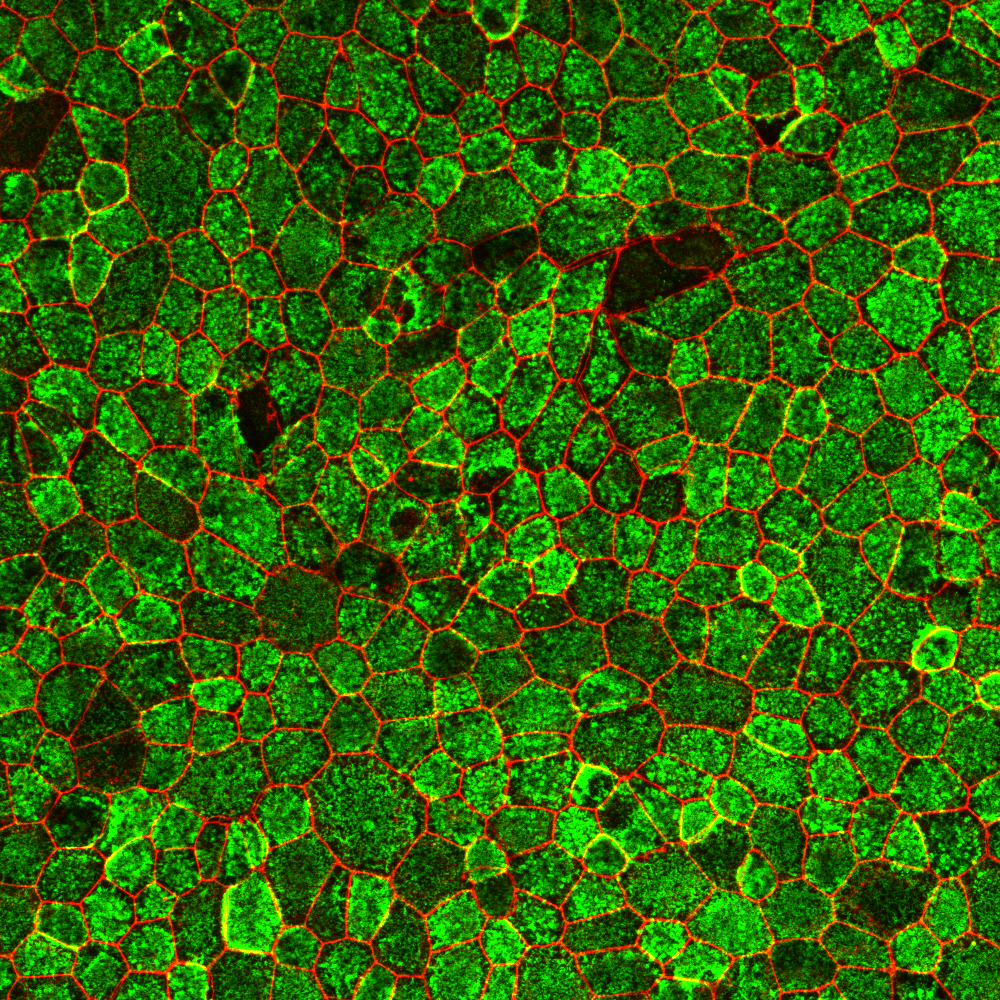

Supplement: Supplementary file 4 — Source data Fig. 2 [file 44319_2025_475_MOESM4_ESM.zip › Figure2/2B/iRPE_Ezrin+ZO-1_RGB.tif]

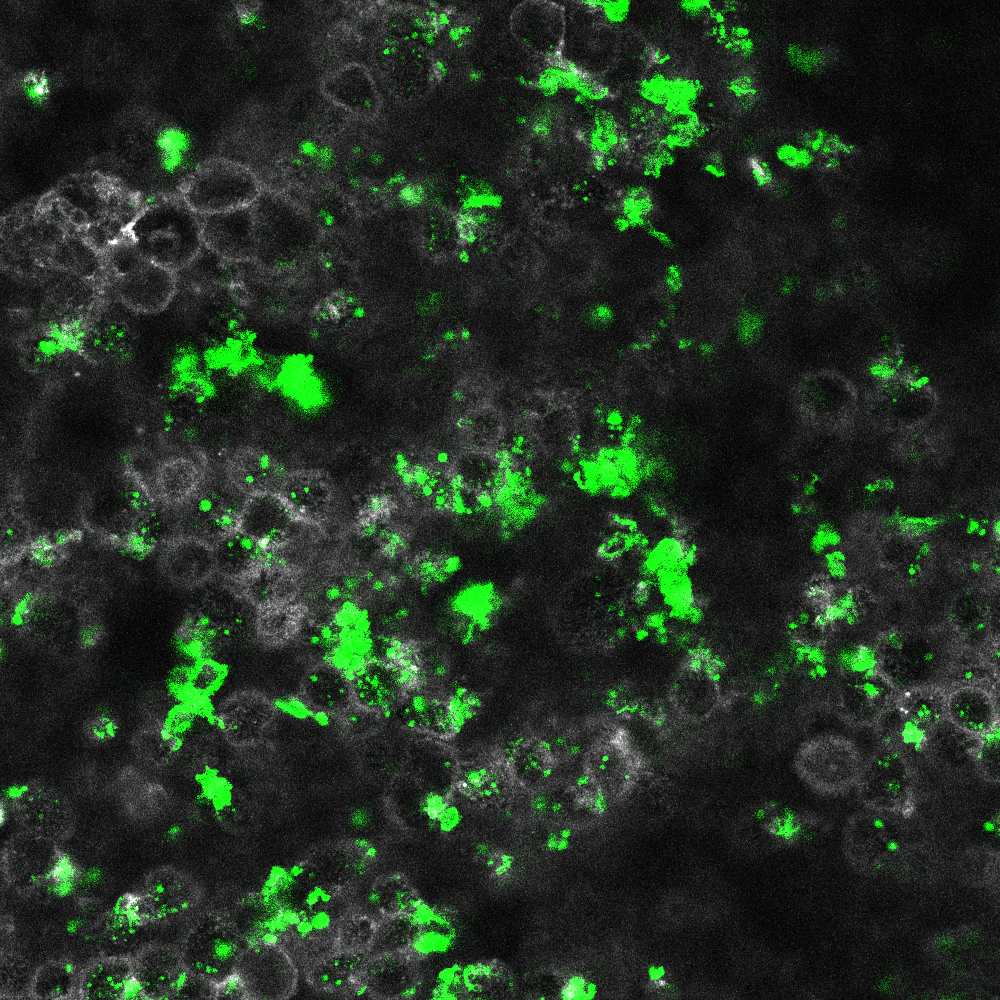

Supplement: Supplementary file 5 — Source data Fig. 3 [file 44319_2025_475_MOESM5_ESM.zip › Figure3/3C/iRPE_LN332_20ug_POS_bound.tif]

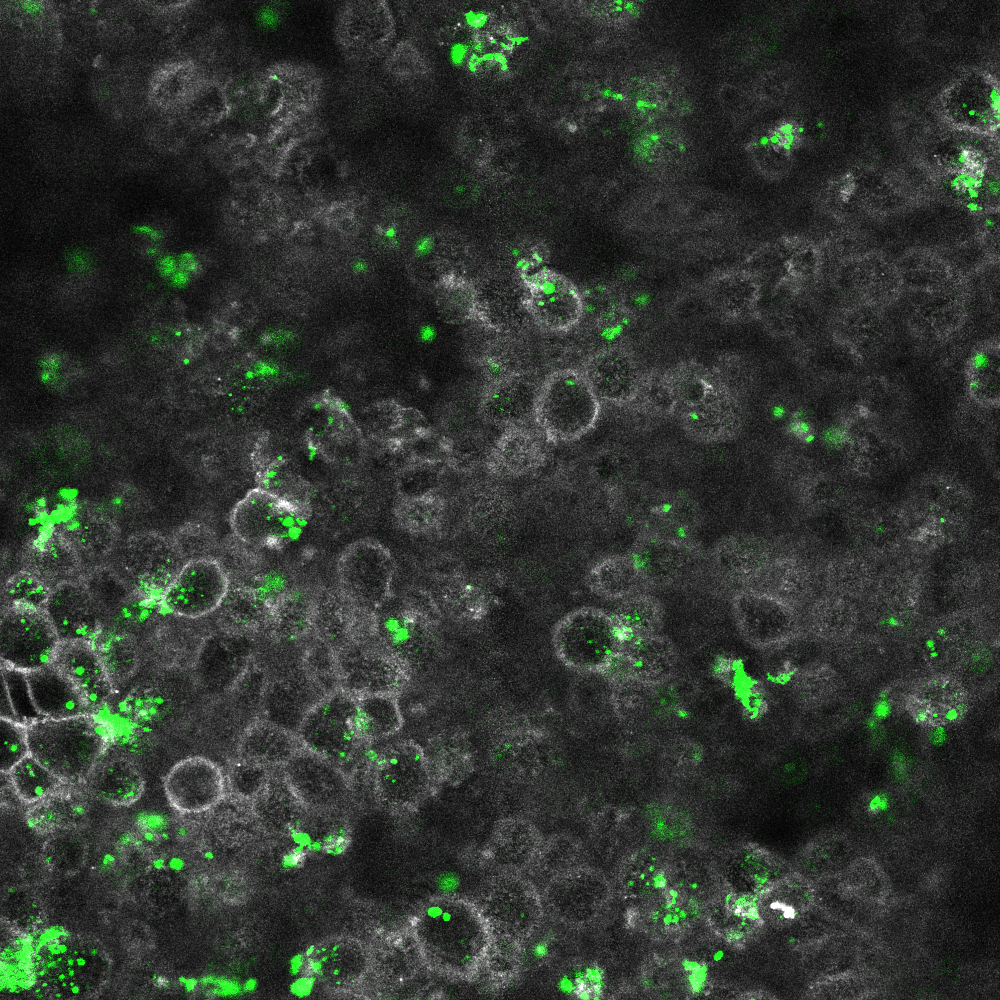

Supplement: Supplementary file 5 — Source data Fig. 3 [file 44319_2025_475_MOESM5_ESM.zip › Figure3/3C/iRPE_LN332_5ug_POS_bound.tif]

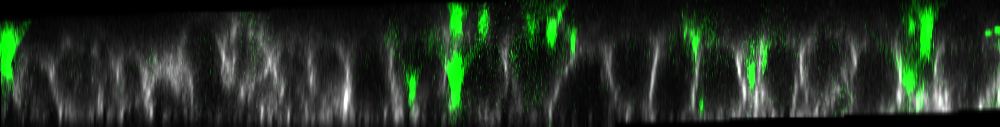

Supplement: Supplementary file 5 — Source data Fig. 3 [file 44319_2025_475_MOESM5_ESM.zip › Figure3/3C/iRPE_LN511_20ug_orthogonal.tif]

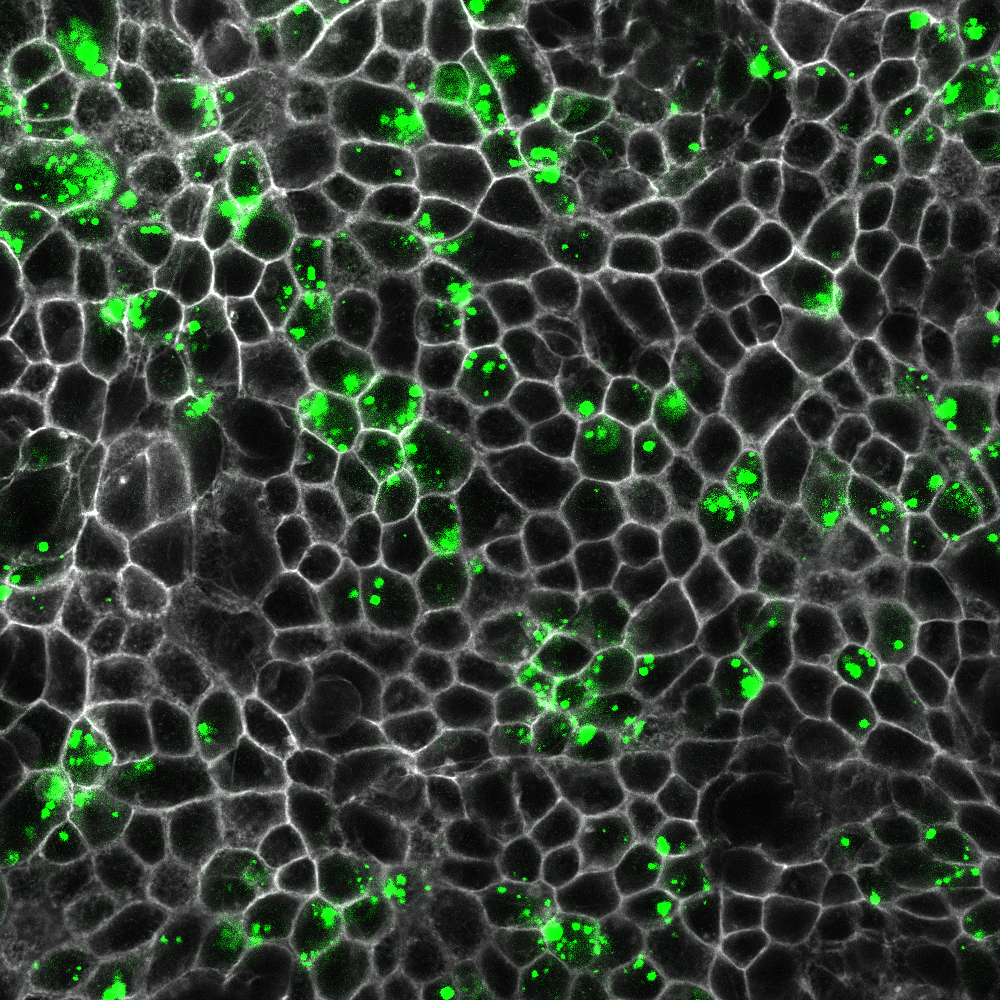

Supplement: Supplementary file 5 — Source data Fig. 3 [file 44319_2025_475_MOESM5_ESM.zip › Figure3/3C/iRPE_LN511_20ug_POS_internilised.tif]

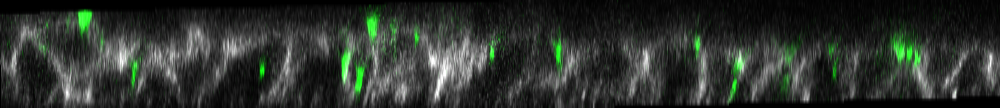

Supplement: Supplementary file 5 — Source data Fig. 3 [file 44319_2025_475_MOESM5_ESM.zip › Figure3/3C/iRPE_LN332_5ug_POS_orthogonal.tif]

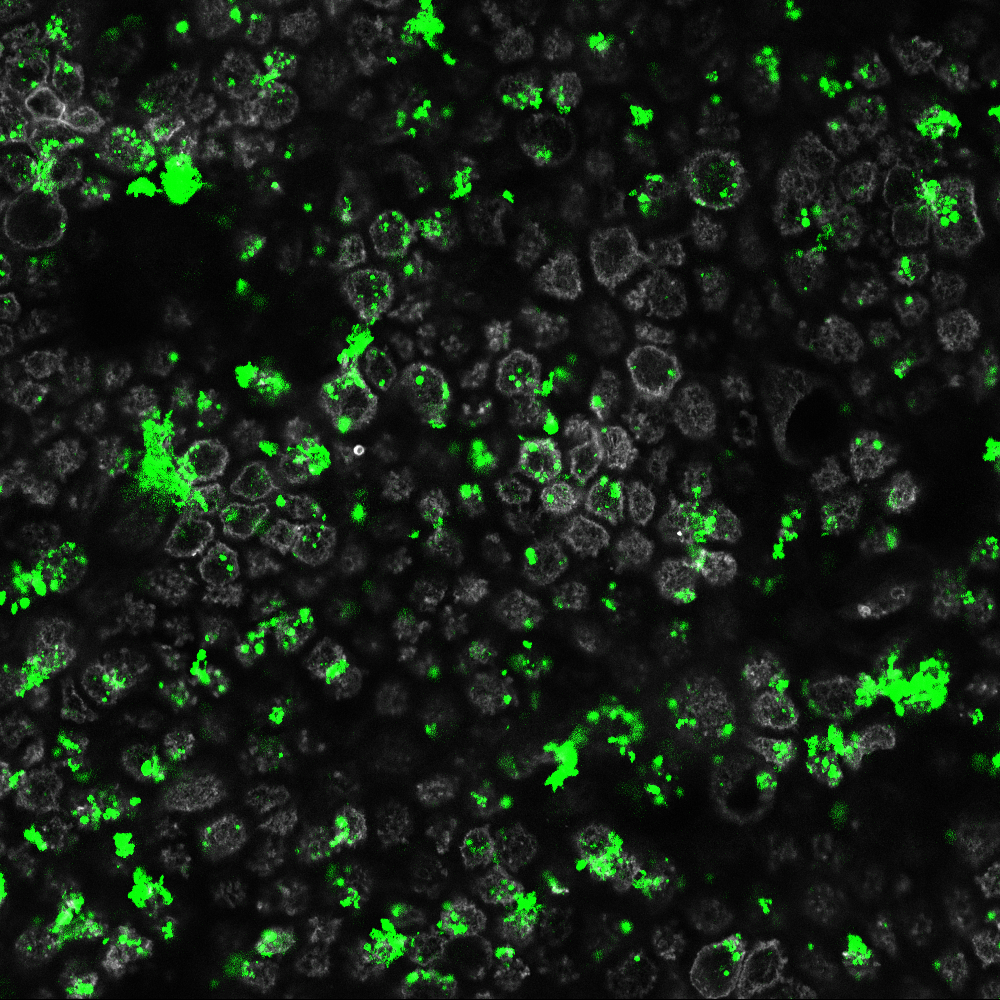

Supplement: Supplementary file 5 — Source data Fig. 3 [file 44319_2025_475_MOESM5_ESM.zip › Figure3/3C/iRPE_LN511_5ug_POS_bound.tif]

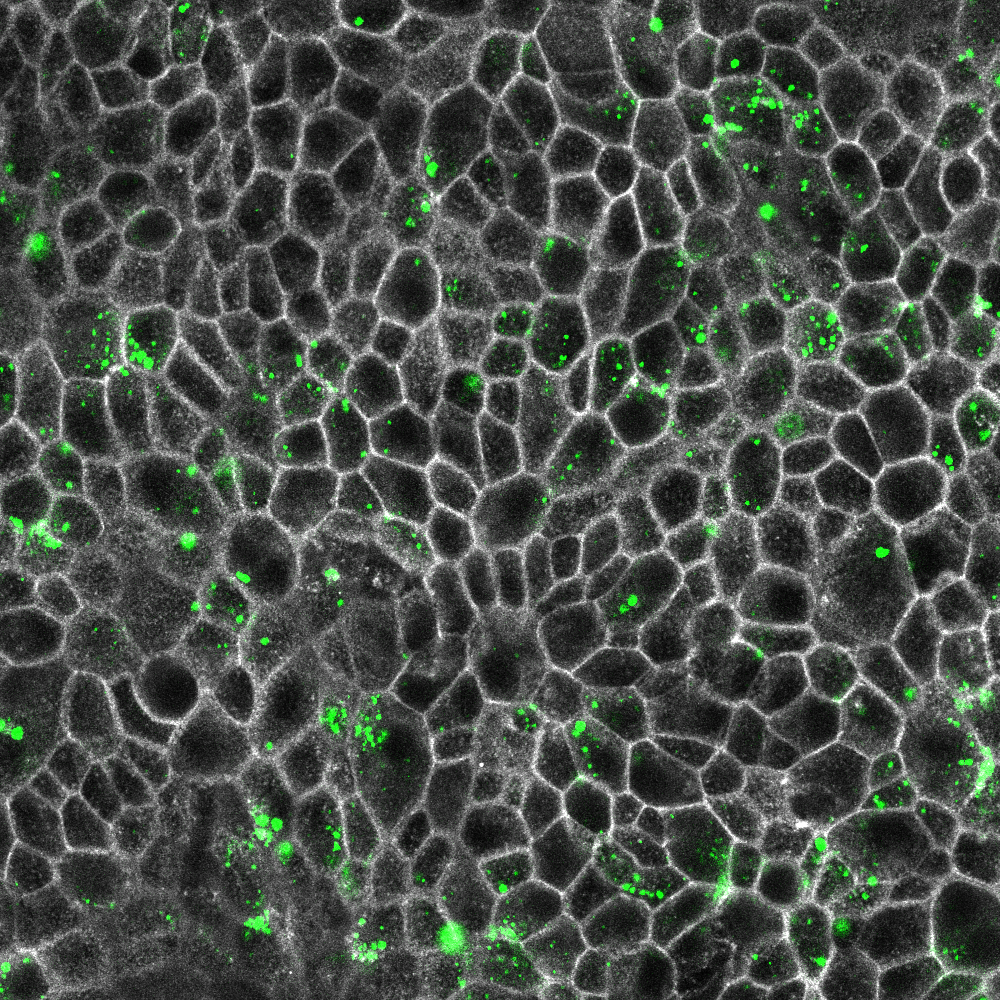

Supplement: Supplementary file 5 — Source data Fig. 3 [file 44319_2025_475_MOESM5_ESM.zip › Figure3/3C/iRPE_LN332_5ug_POS_internalised.tif]

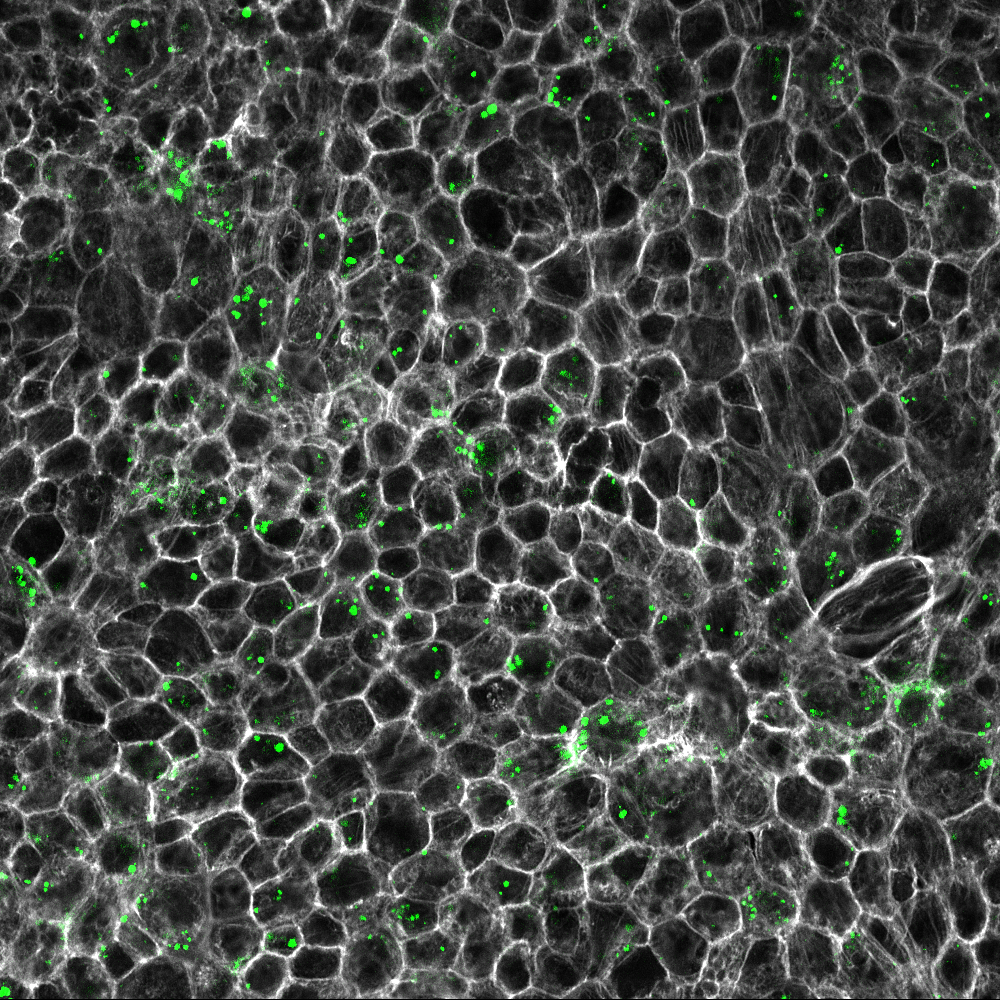

Supplement: Supplementary file 5 — Source data Fig. 3 [file 44319_2025_475_MOESM5_ESM.zip › Figure3/3C/iRPE_LN511_5ug_POS_internilised.tif]

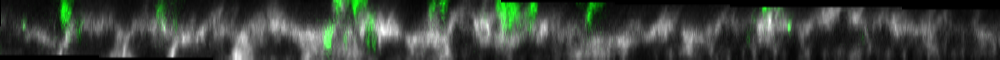

Supplement: Supplementary file 5 — Source data Fig. 3 [file 44319_2025_475_MOESM5_ESM.zip › Figure3/3C/iRPE_LN511_5ug_POS_orthogonal.tif]

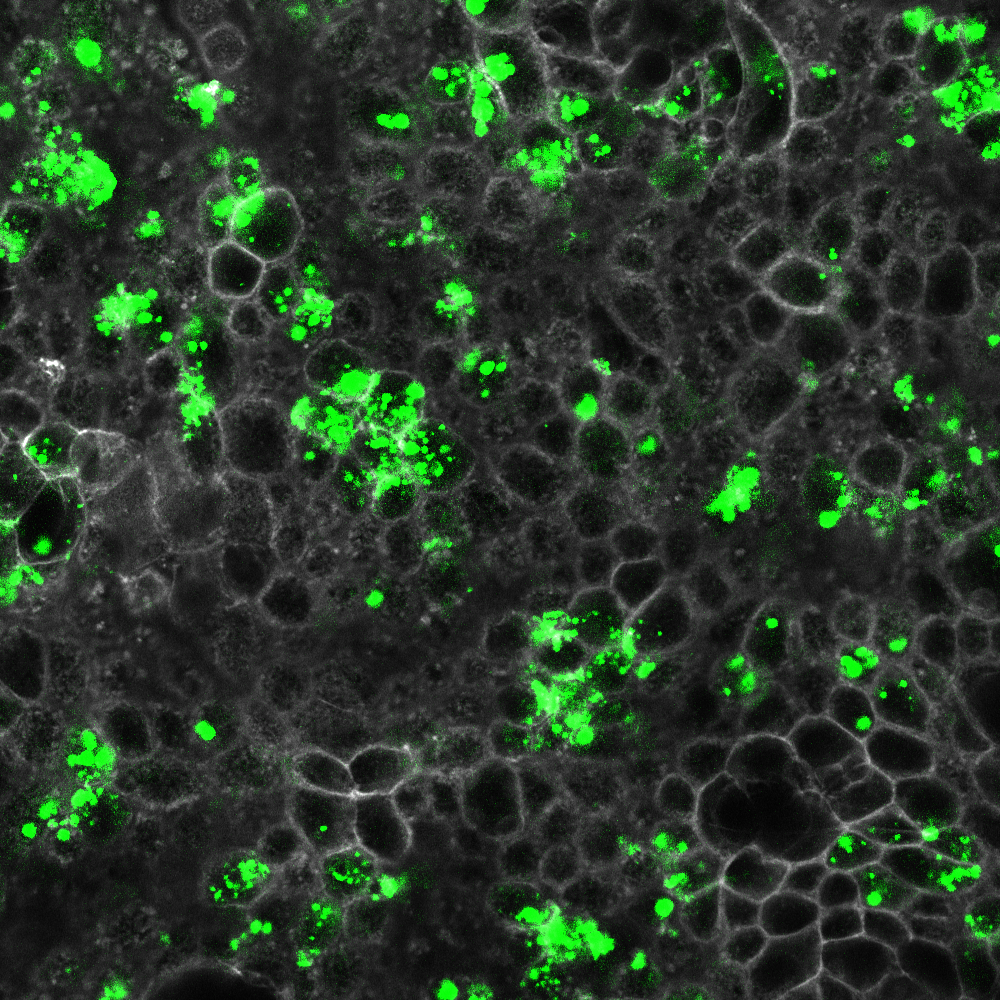

Supplement: Supplementary file 5 — Source data Fig. 3 [file 44319_2025_475_MOESM5_ESM.zip › Figure3/3C/iRPE_LN511_20ug_POS_bound.tif]

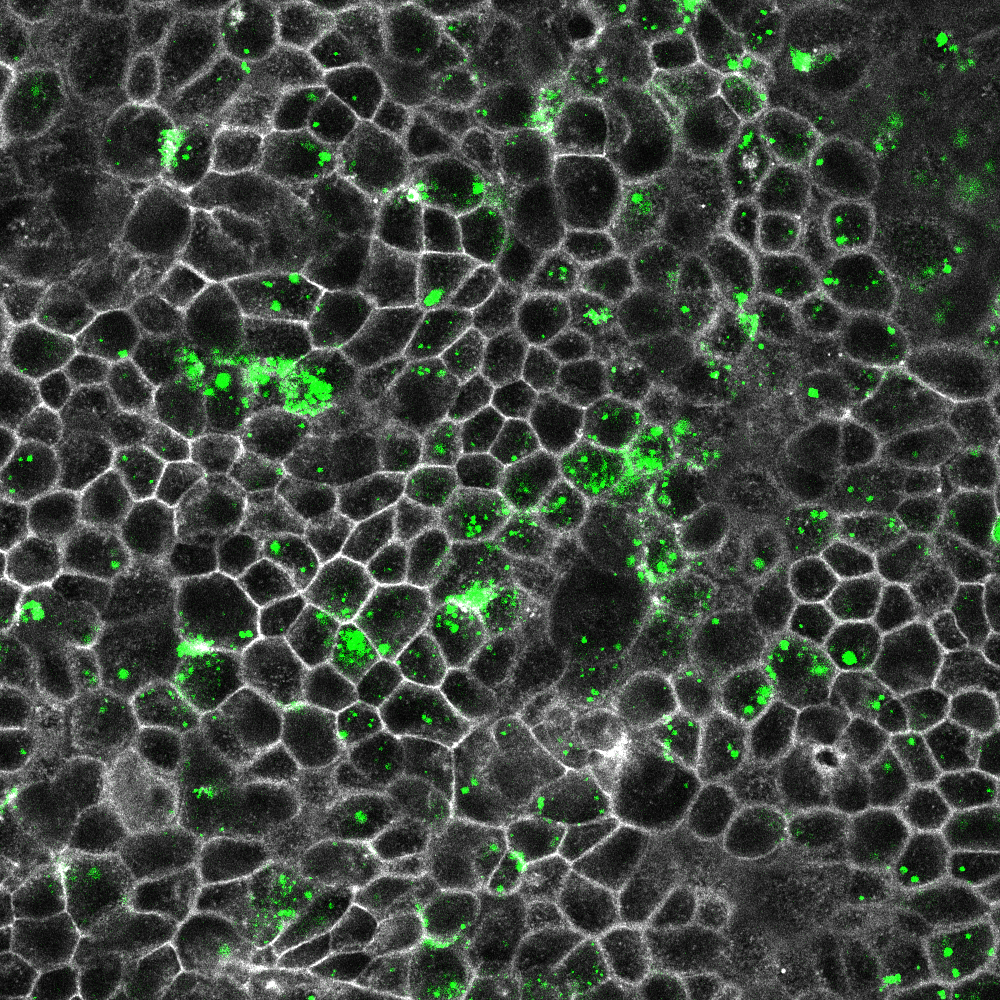

Supplement: Supplementary file 5 — Source data Fig. 3 [file 44319_2025_475_MOESM5_ESM.zip › Figure3/3C/iRPE_LN332_20ug_POS_internilised.tif]

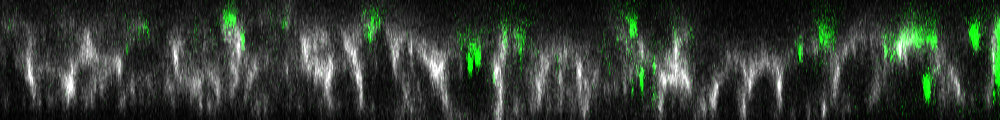

Supplement: Supplementary file 5 — Source data Fig. 3 [file 44319_2025_475_MOESM5_ESM.zip › Figure3/3C/iRPE_LN332_20ug_POS_orthogonal.tif]

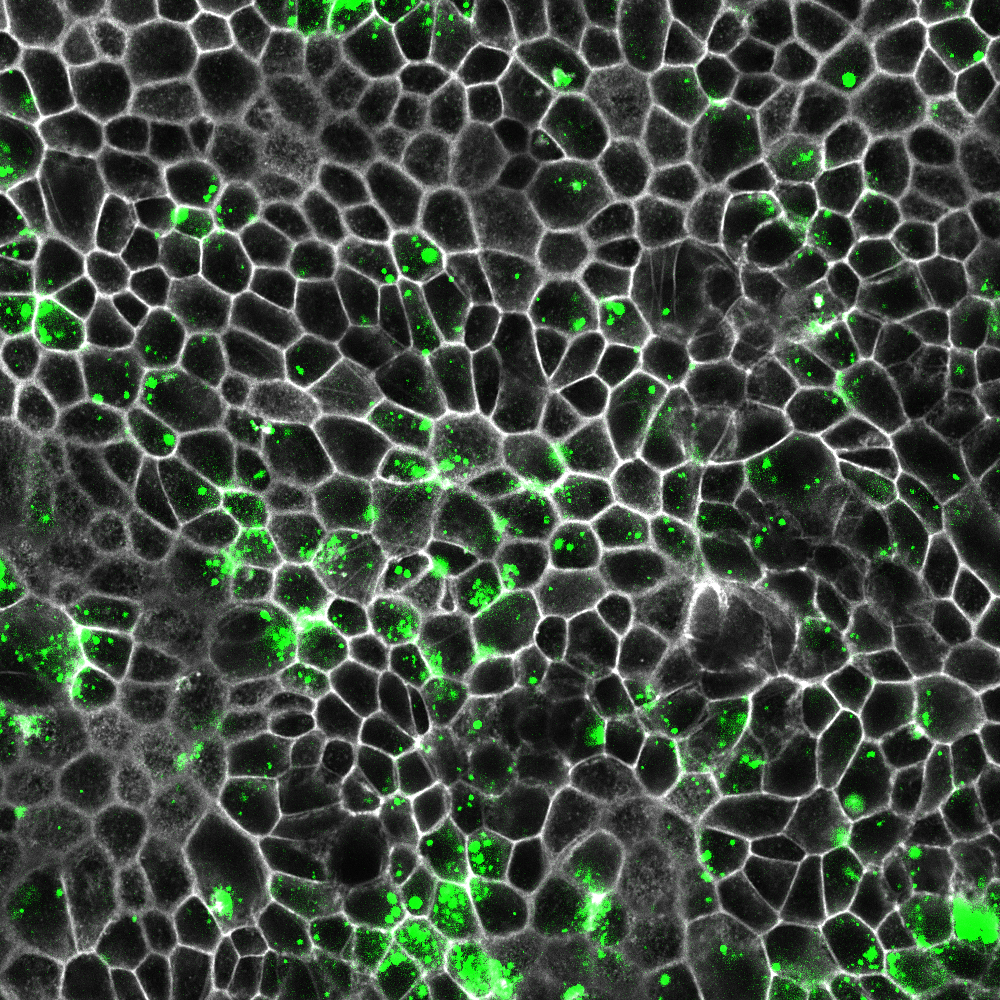

Supplement: Supplementary file 5 — Source data Fig. 3 [file 44319_2025_475_MOESM5_ESM.zip › Figure3/3J/iRPE_LN511_ROCKContr_POS_internilised.tif]

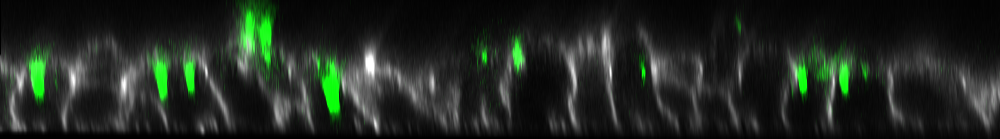

Supplement: Supplementary file 5 — Source data Fig. 3 [file 44319_2025_475_MOESM5_ESM.zip › Figure3/3J/iRPE_LN511_ROCK_POS_orthogonal.tif]

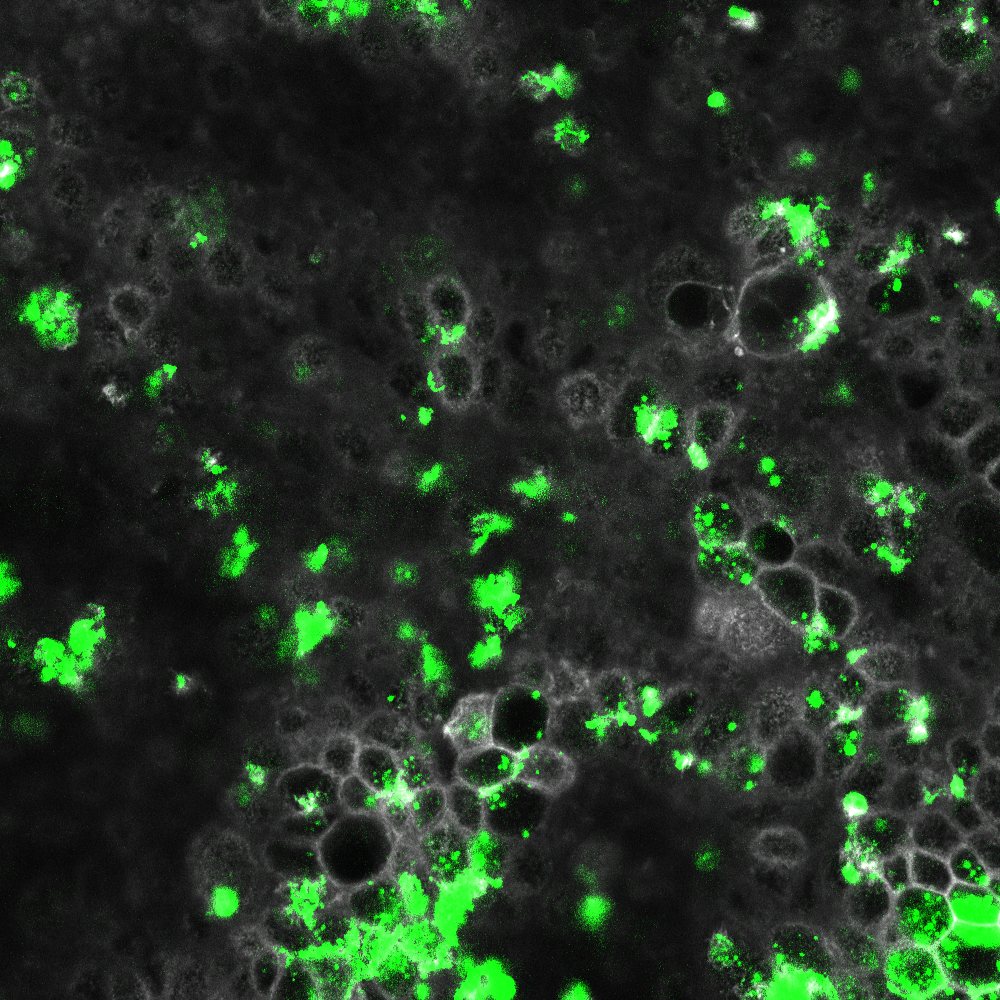

Supplement: Supplementary file 5 — Source data Fig. 3 [file 44319_2025_475_MOESM5_ESM.zip › Figure3/3J/iRPE_LN511_ROCKContr_POS_bound.tif]

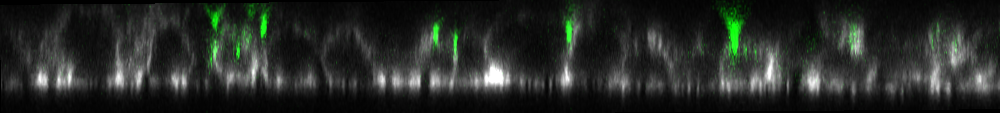

Supplement: Supplementary file 5 — Source data Fig. 3 [file 44319_2025_475_MOESM5_ESM.zip › Figure3/3J/iRPE_LN511_RhoA_POS_orthogonal.tif]

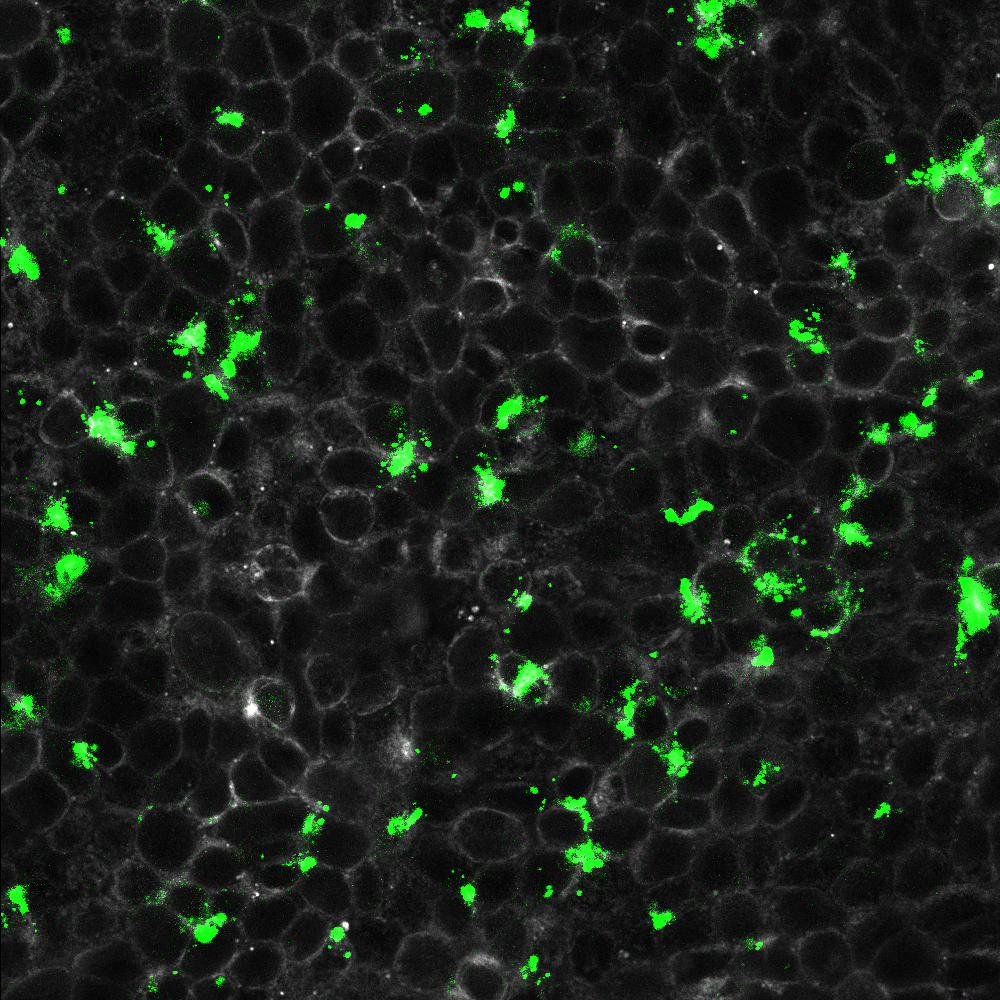

Supplement: Supplementary file 5 — Source data Fig. 3 [file 44319_2025_475_MOESM5_ESM.zip › Figure3/3J/iRPE_LN511_RhoA_POS_bound.tif]

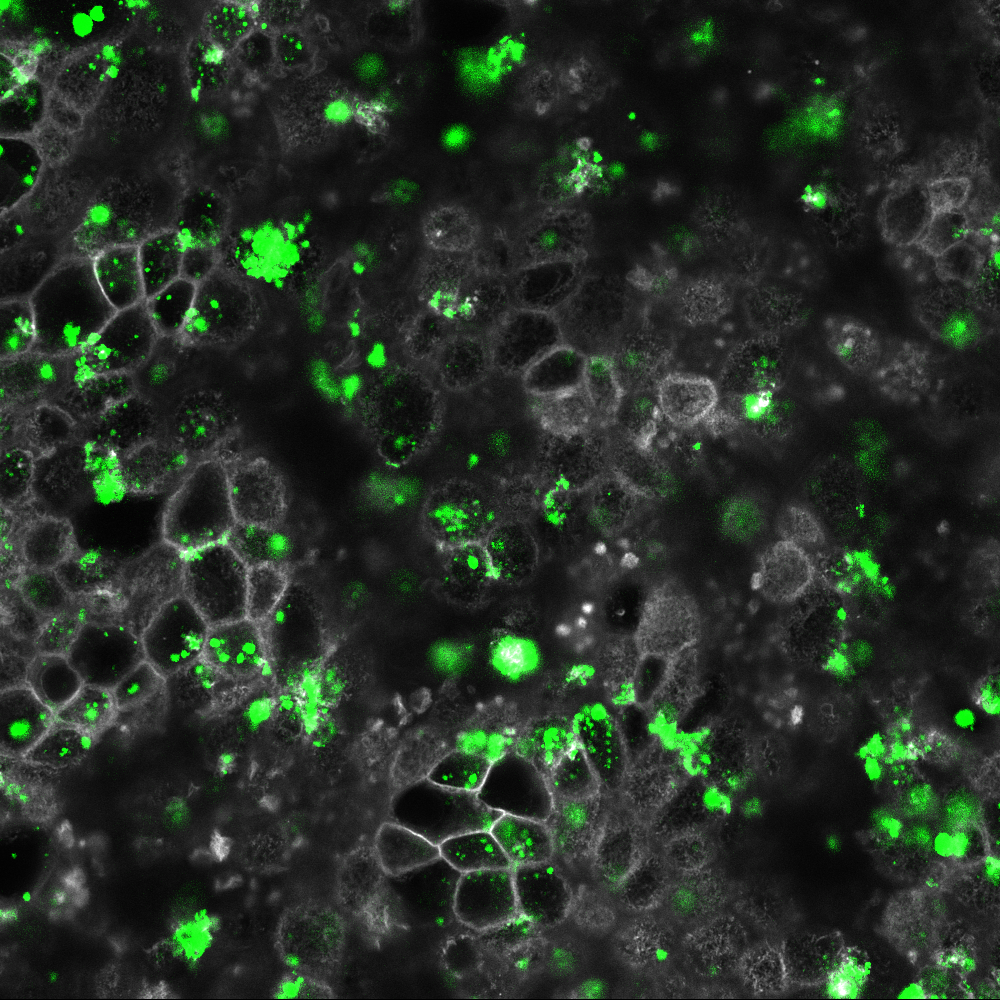

Supplement: Supplementary file 5 — Source data Fig. 3 [file 44319_2025_475_MOESM5_ESM.zip › Figure3/3J/iRPE_LN511_ROCK_POS_bound.tif]

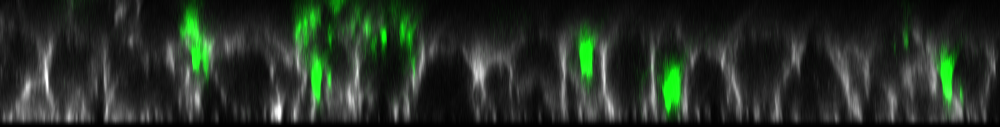

Supplement: Supplementary file 5 — Source data Fig. 3 [file 44319_2025_475_MOESM5_ESM.zip › Figure3/3J/iRPE_LN511_RhoACntr_POS_orthogonal.tif]

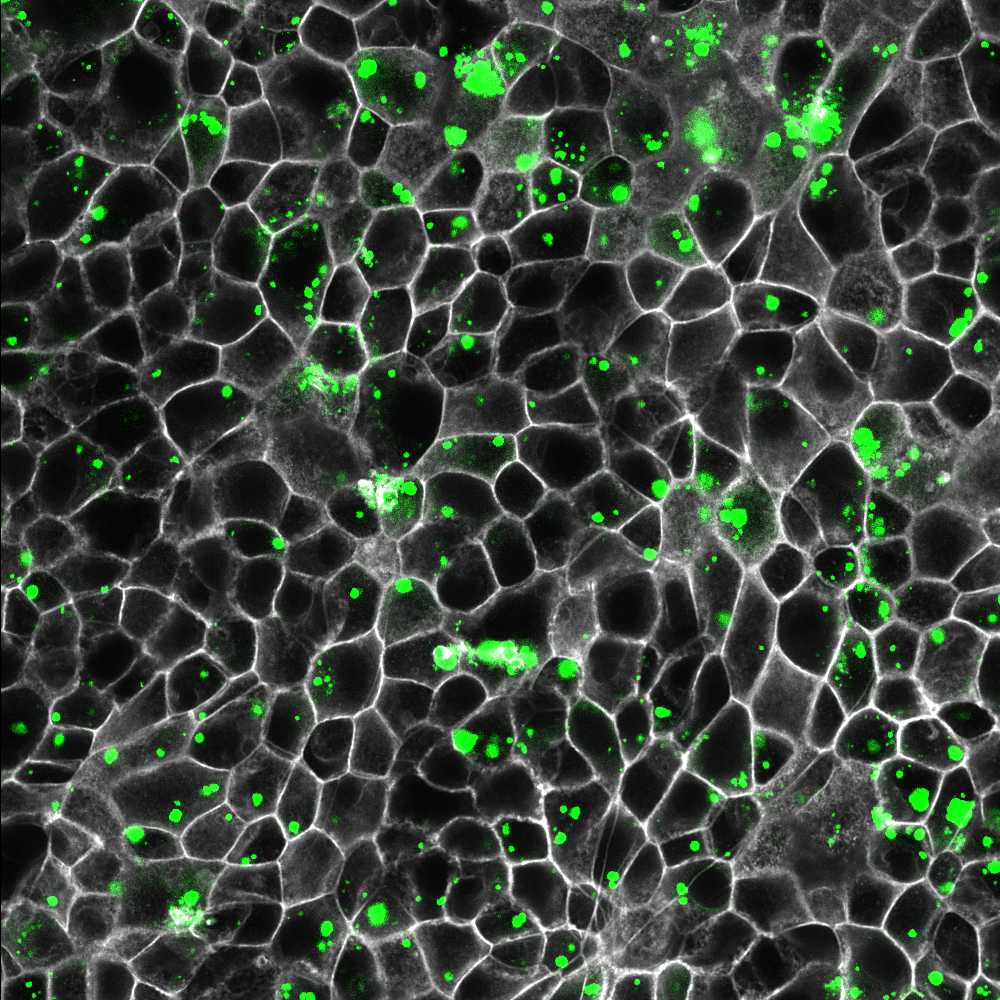

Supplement: Supplementary file 5 — Source data Fig. 3 [file 44319_2025_475_MOESM5_ESM.zip › Figure3/3J/iRPE_LN511_ROCK_POS_internilised.tif]

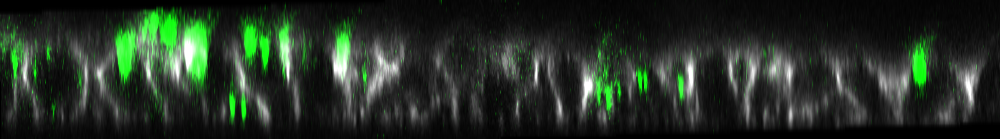

Supplement: Supplementary file 5 — Source data Fig. 3 [file 44319_2025_475_MOESM5_ESM.zip › Figure3/3J/iRPE_LN511_ROCKContr_POS_orthogonal.tif]

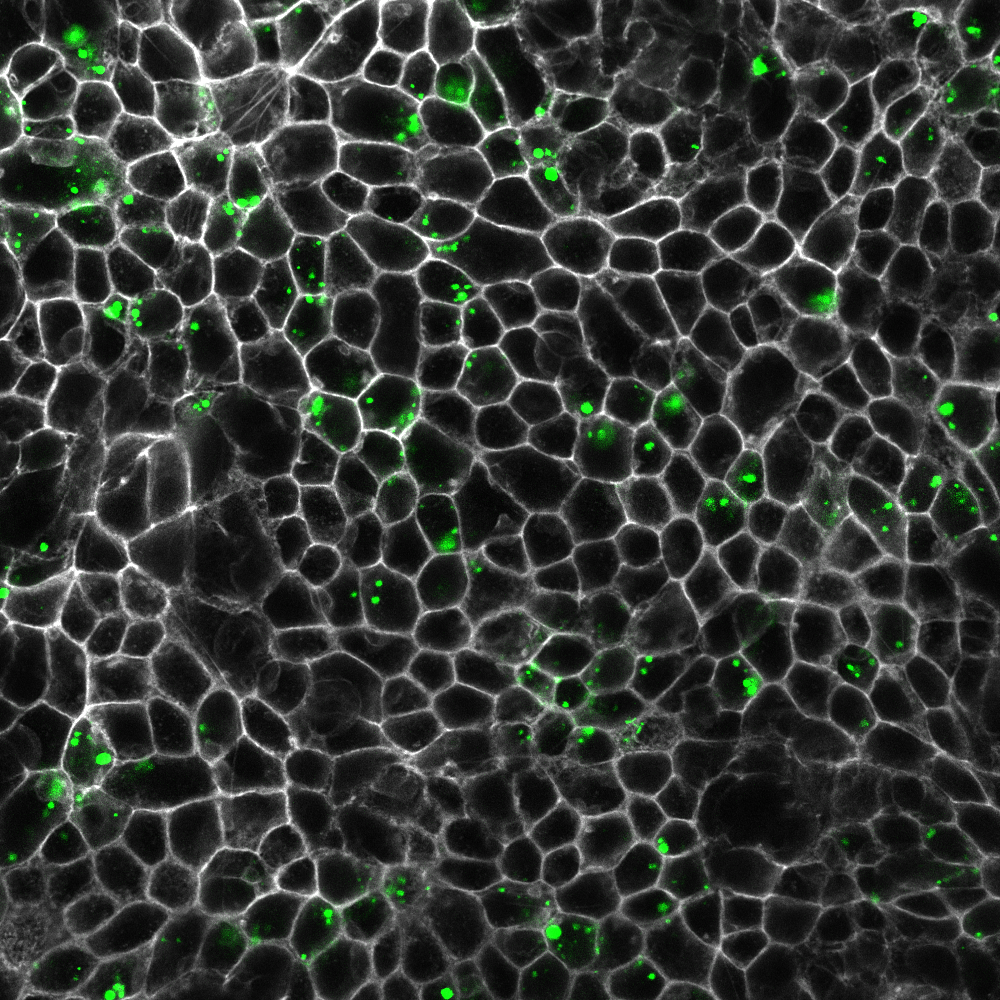

Supplement: Supplementary file 5 — Source data Fig. 3 [file 44319_2025_475_MOESM5_ESM.zip › Figure3/3J/iRPE_LN511_RhoACntr_POS_internilised.tif]

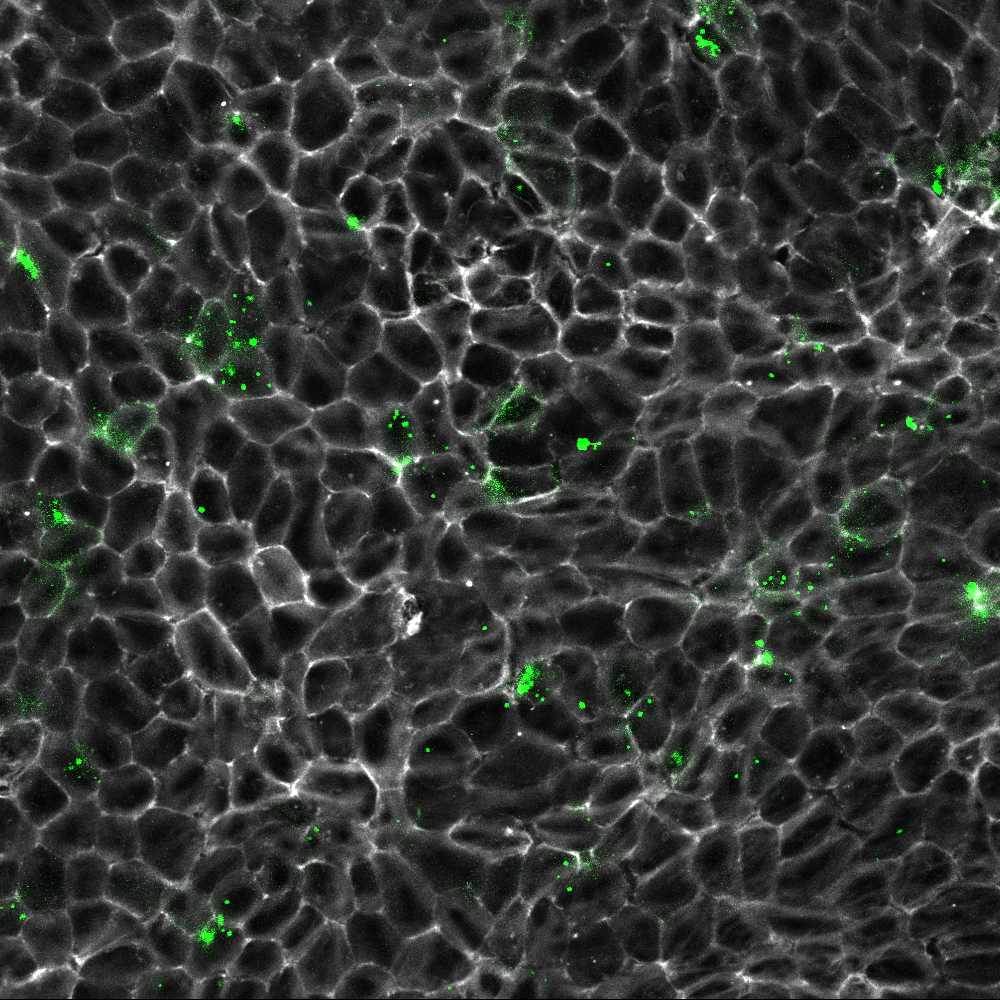

Supplement: Supplementary file 5 — Source data Fig. 3 [file 44319_2025_475_MOESM5_ESM.zip › Figure3/3J/iRPE_LN511_RhoA_POS_internilised.tif]

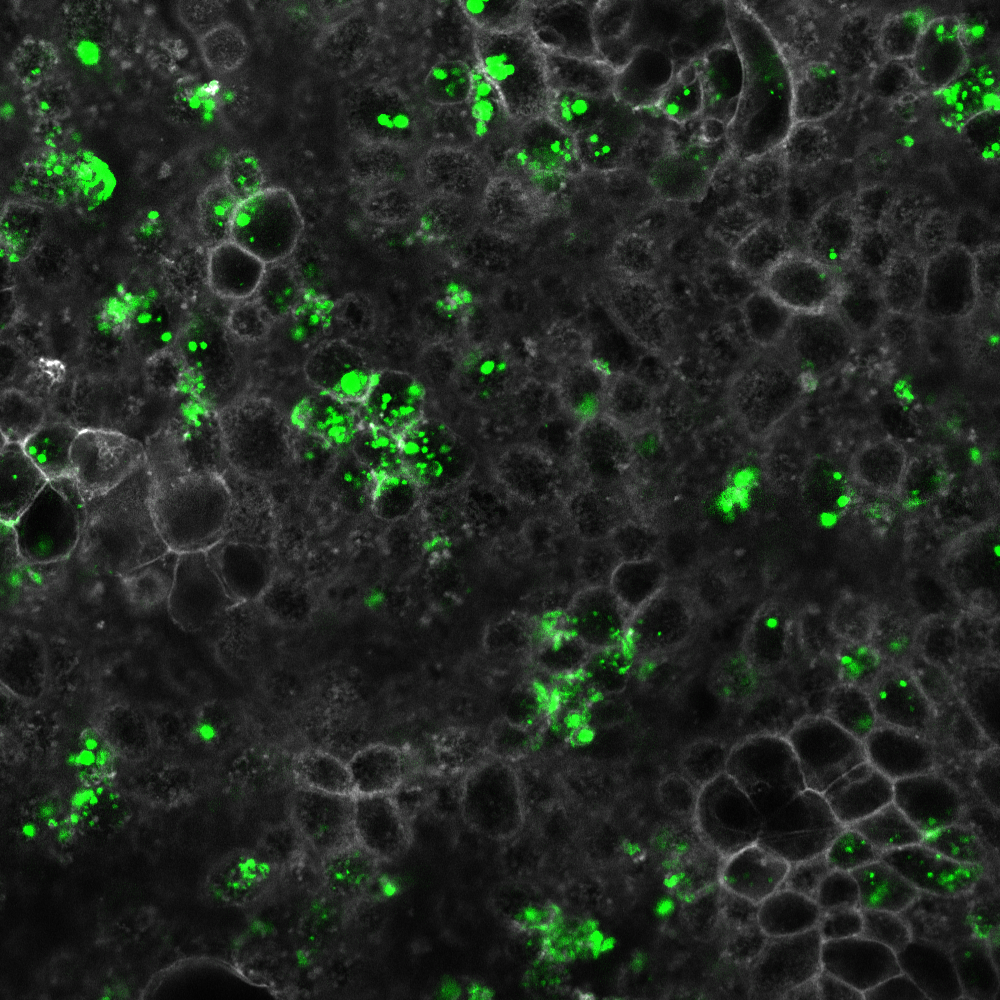

Supplement: Supplementary file 5 — Source data Fig. 3 [file 44319_2025_475_MOESM5_ESM.zip › Figure3/3J/iRPE_LN511_RhoACntr_POS_bound.tif]

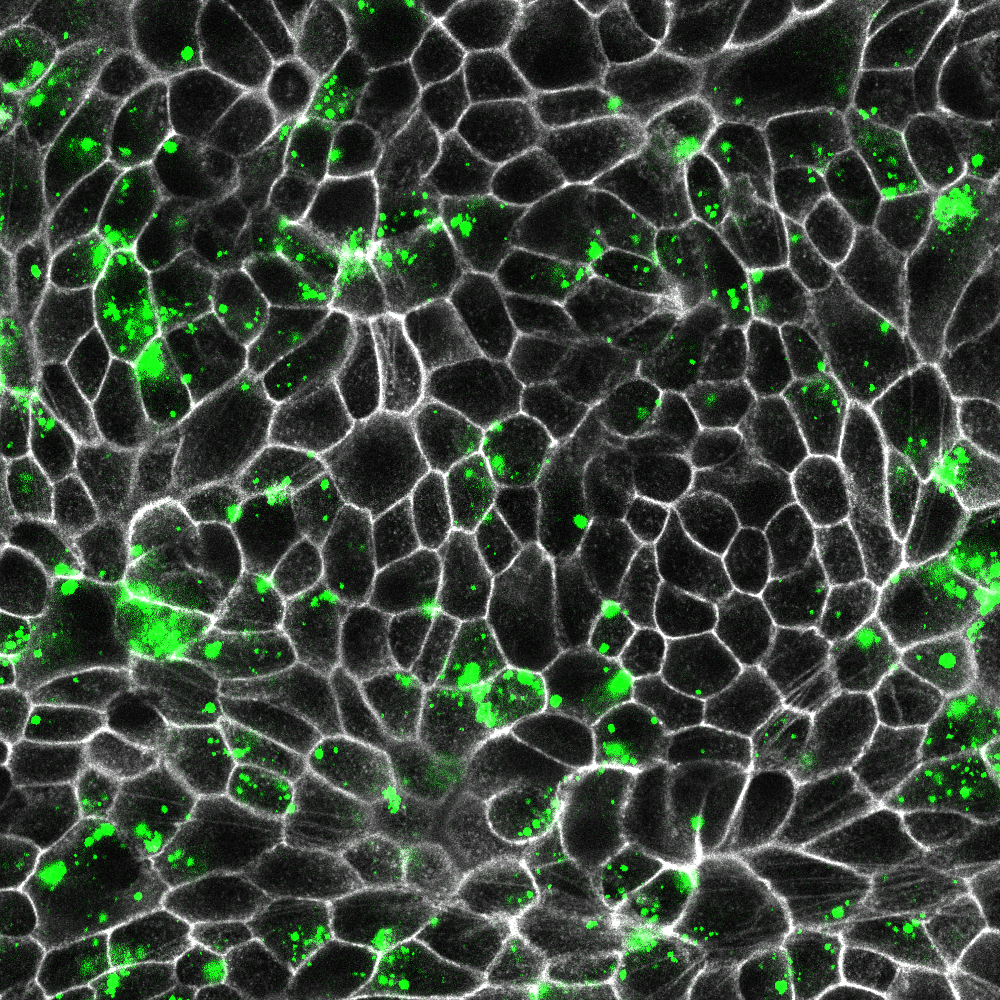

Supplement: Supplementary file 5 — Source data Fig. 3 [file 44319_2025_475_MOESM5_ESM.zip › Figure3/3G/iRPE_LN511_18kPa_POS_internalised.tif]

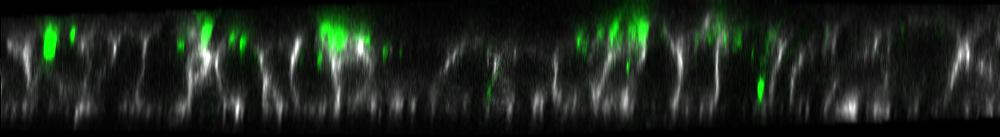

Supplement: Supplementary file 5 — Source data Fig. 3 [file 44319_2025_475_MOESM5_ESM.zip › Figure3/3G/iRPE_VN_4kPa_POS_orthogonal.tif]

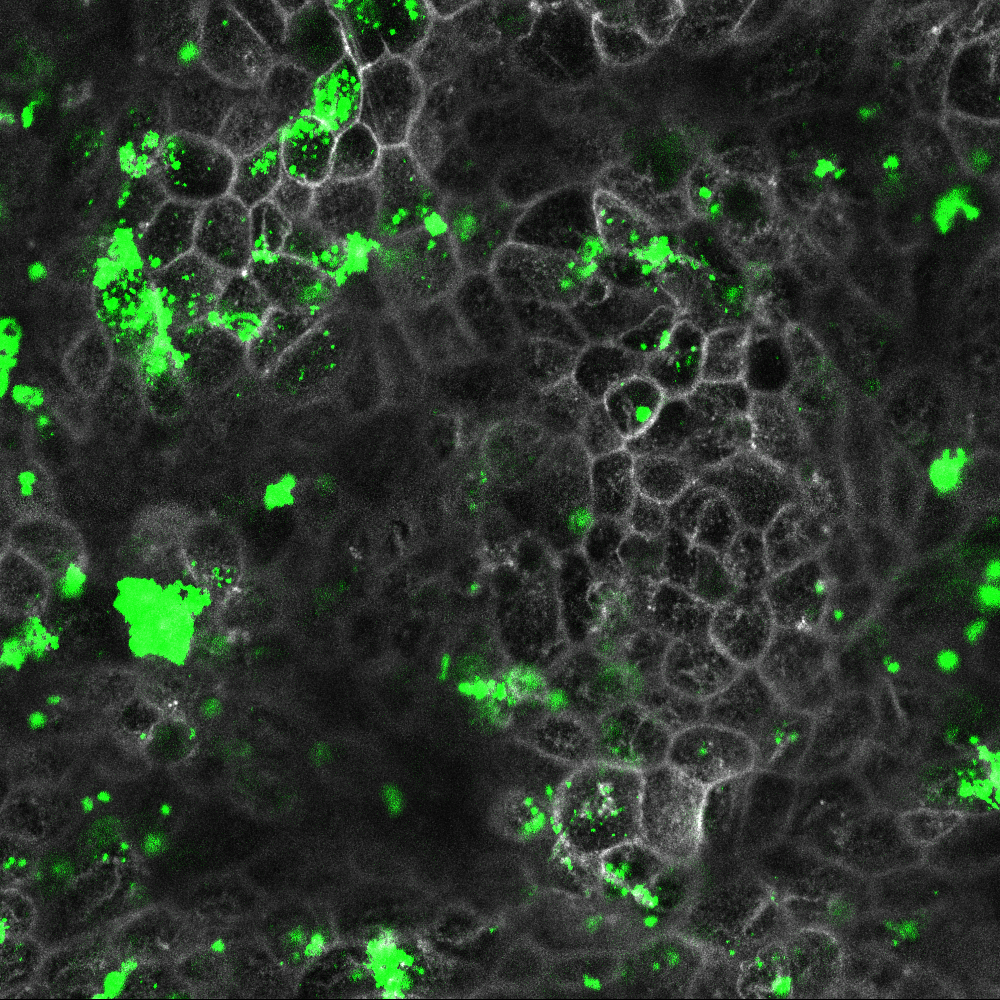

Supplement: Supplementary file 5 — Source data Fig. 3 [file 44319_2025_475_MOESM5_ESM.zip › Figure3/3G/iRPE_LN511_18kPa_POS_bound.tif]

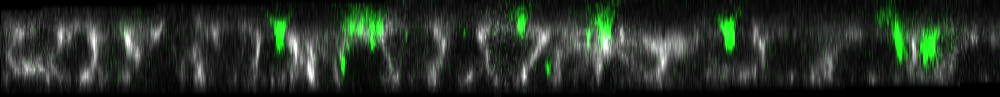

Supplement: Supplementary file 5 — Source data Fig. 3 [file 44319_2025_475_MOESM5_ESM.zip › Figure3/3G/iRPE_LN511_18kPa_POS_orthogonal.tif]

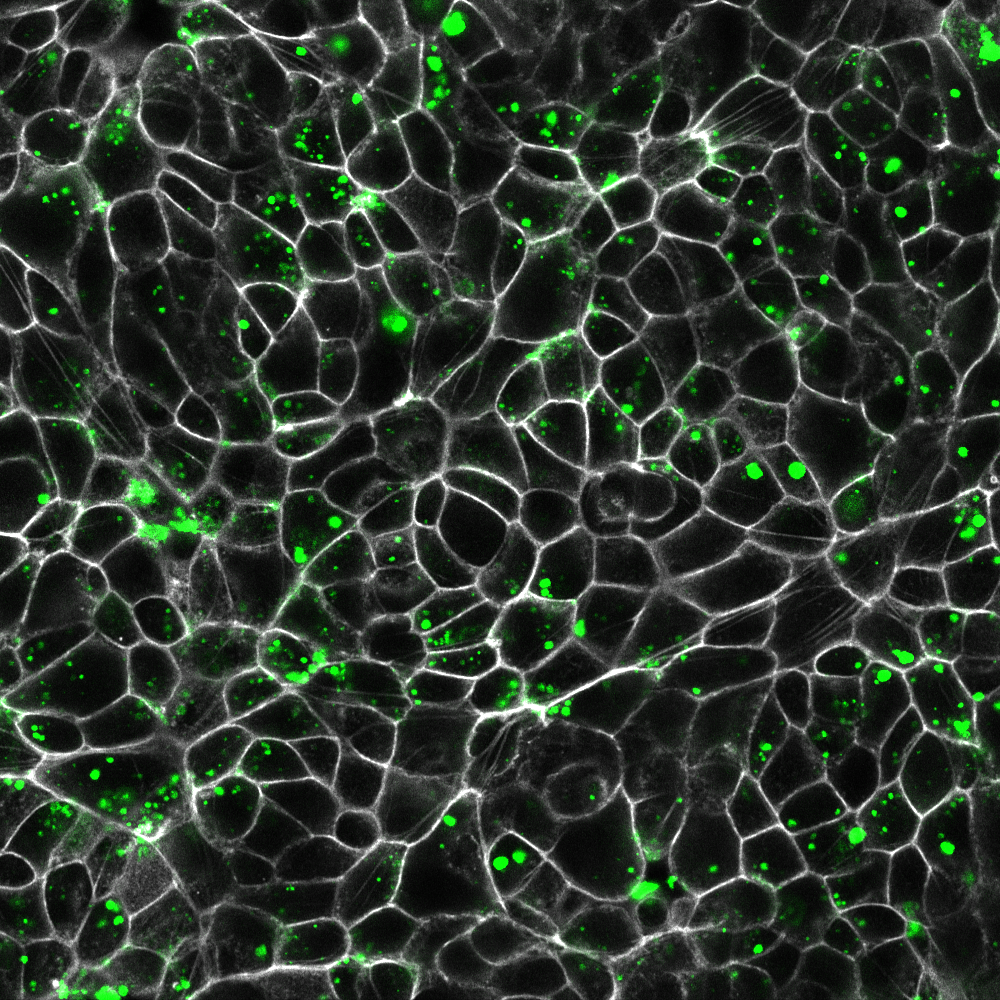

Supplement: Supplementary file 5 — Source data Fig. 3 [file 44319_2025_475_MOESM5_ESM.zip › Figure3/3G/iRPE_VN_4kPa_POS_internalised.tif]

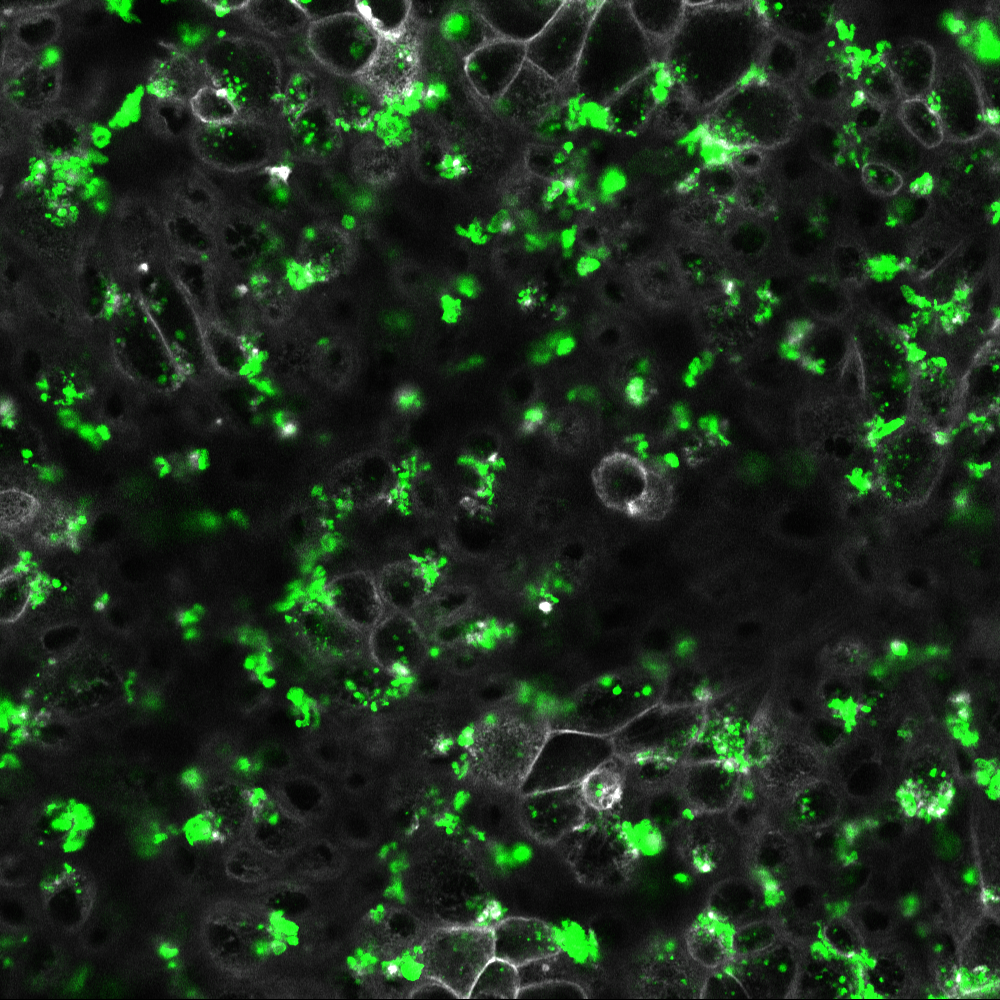

Supplement: Supplementary file 5 — Source data Fig. 3 [file 44319_2025_475_MOESM5_ESM.zip › Figure3/3G/iRPE_VN_4kPa_POS_bound.tif]

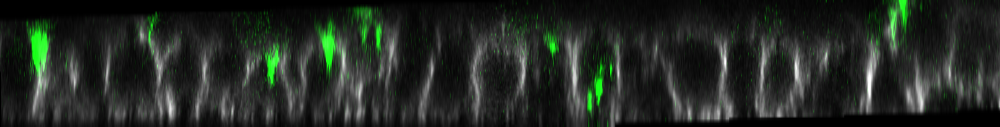

Supplement: Supplementary file 5 — Source data Fig. 3 [file 44319_2025_475_MOESM5_ESM.zip › Figure3/3G/iRPE_LN511_4kPa_POS_orthogonal.tif]

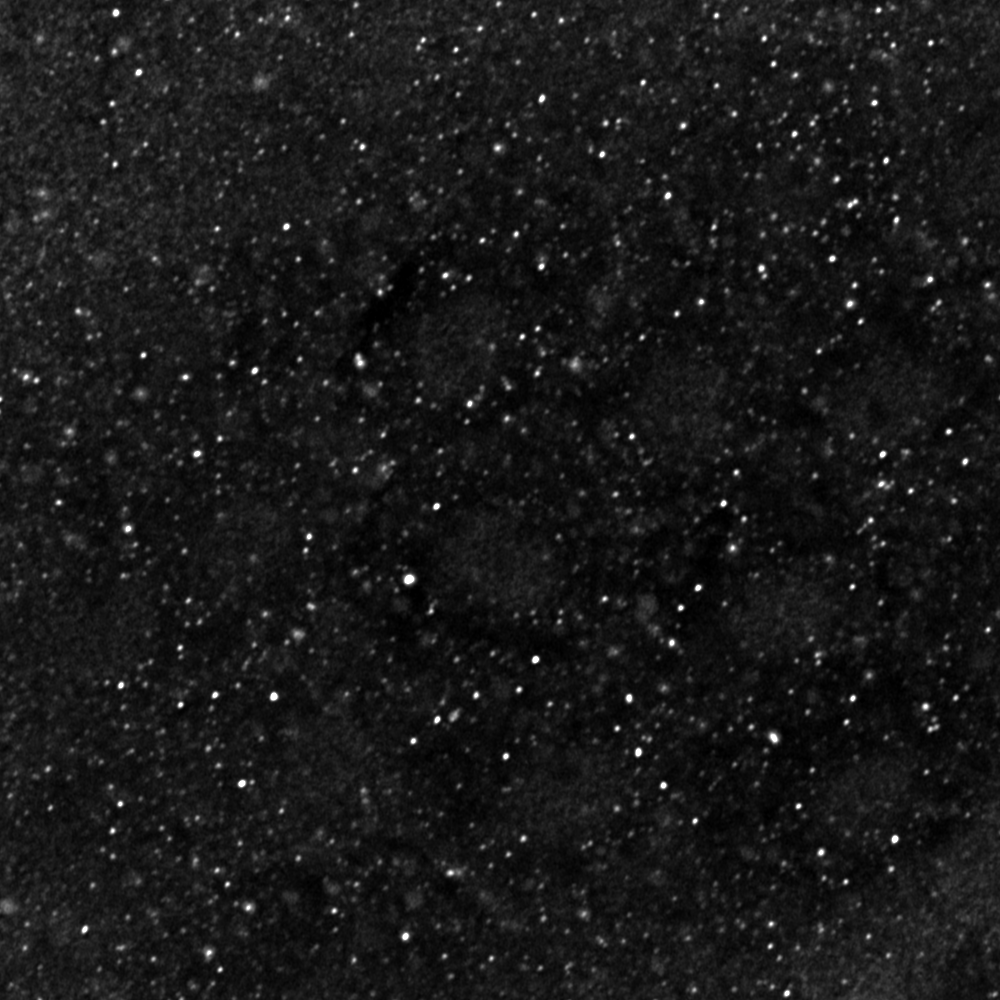

Supplement: Supplementary file 7 — Source data Fig. 5 [file 44319_2025_475_MOESM7_ESM.zip › Figure5/5A/Integrin b1_MidPeriphery.tif]

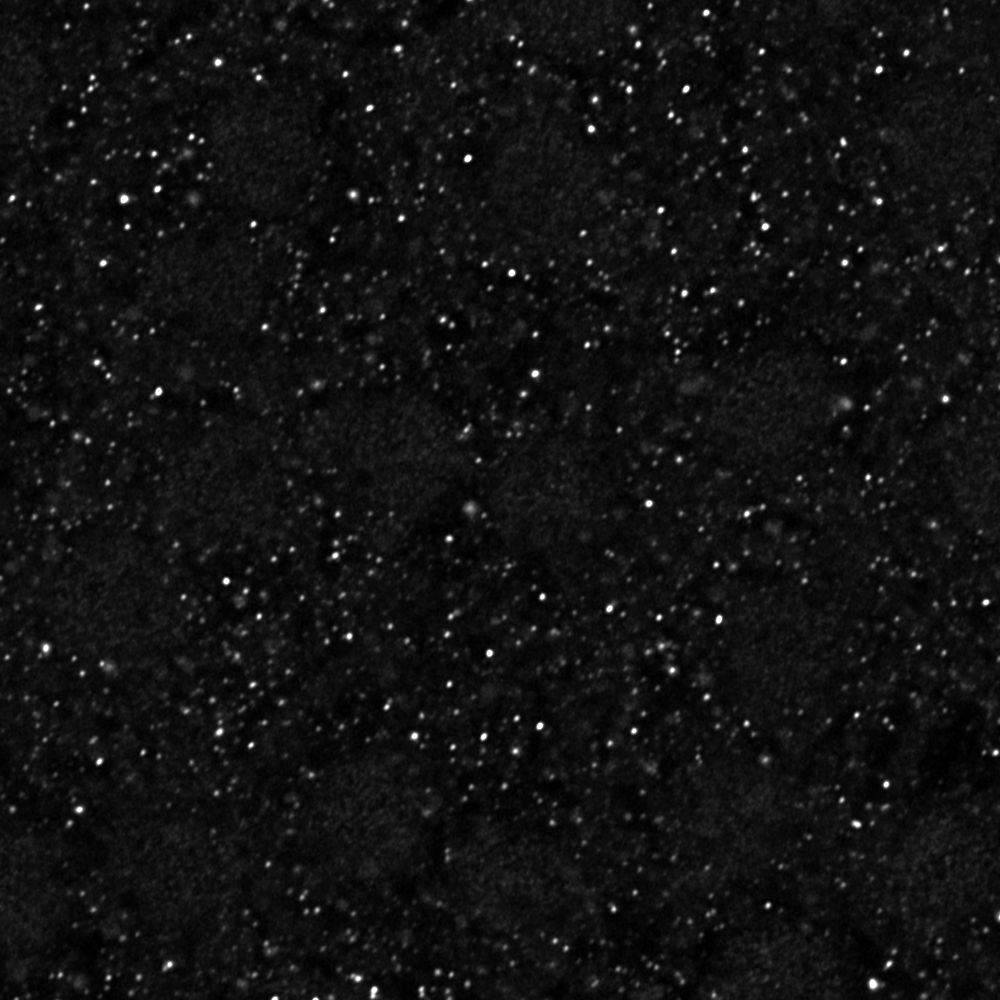

Supplement: Supplementary file 7 — Source data Fig. 5 [file 44319_2025_475_MOESM7_ESM.zip › Figure5/5A/Integrin b1_Centre.tif]

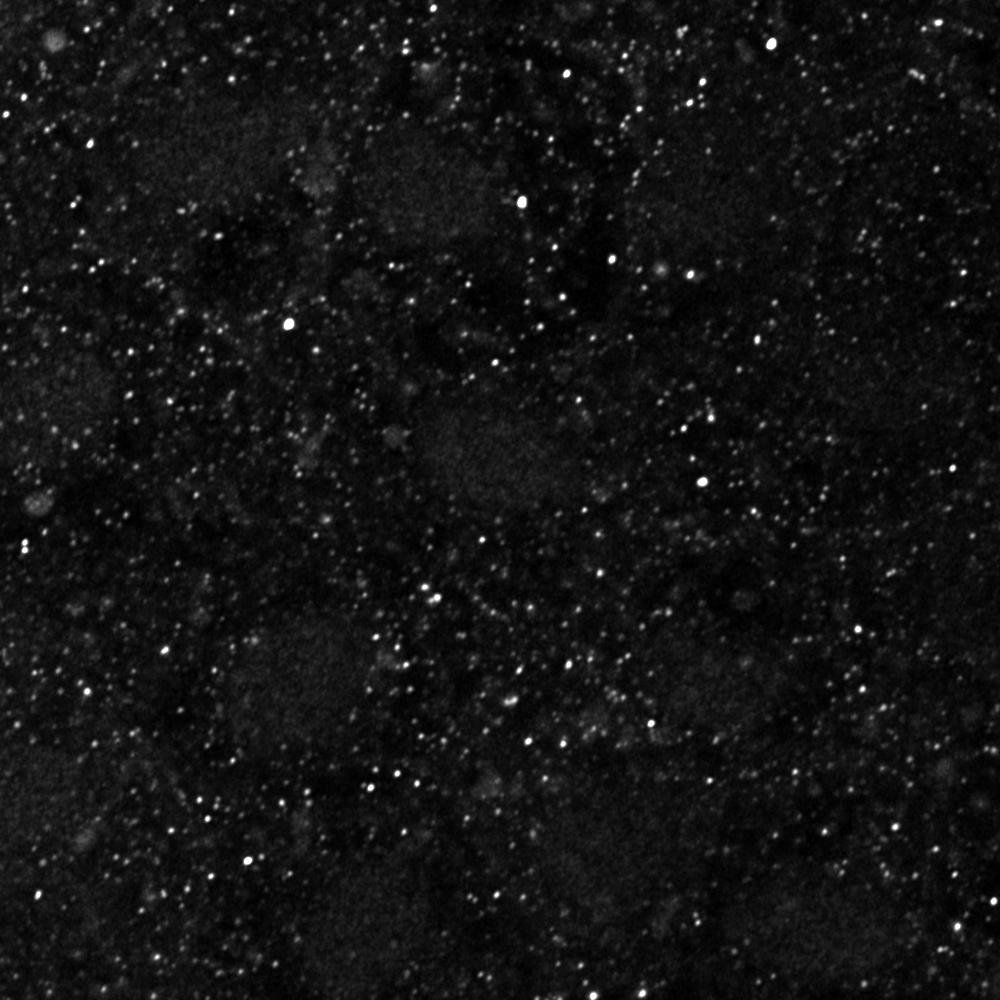

Supplement: Supplementary file 7 — Source data Fig. 5 [file 44319_2025_475_MOESM7_ESM.zip › Figure5/5A/Integrin b1_FarPeriphery.tif]

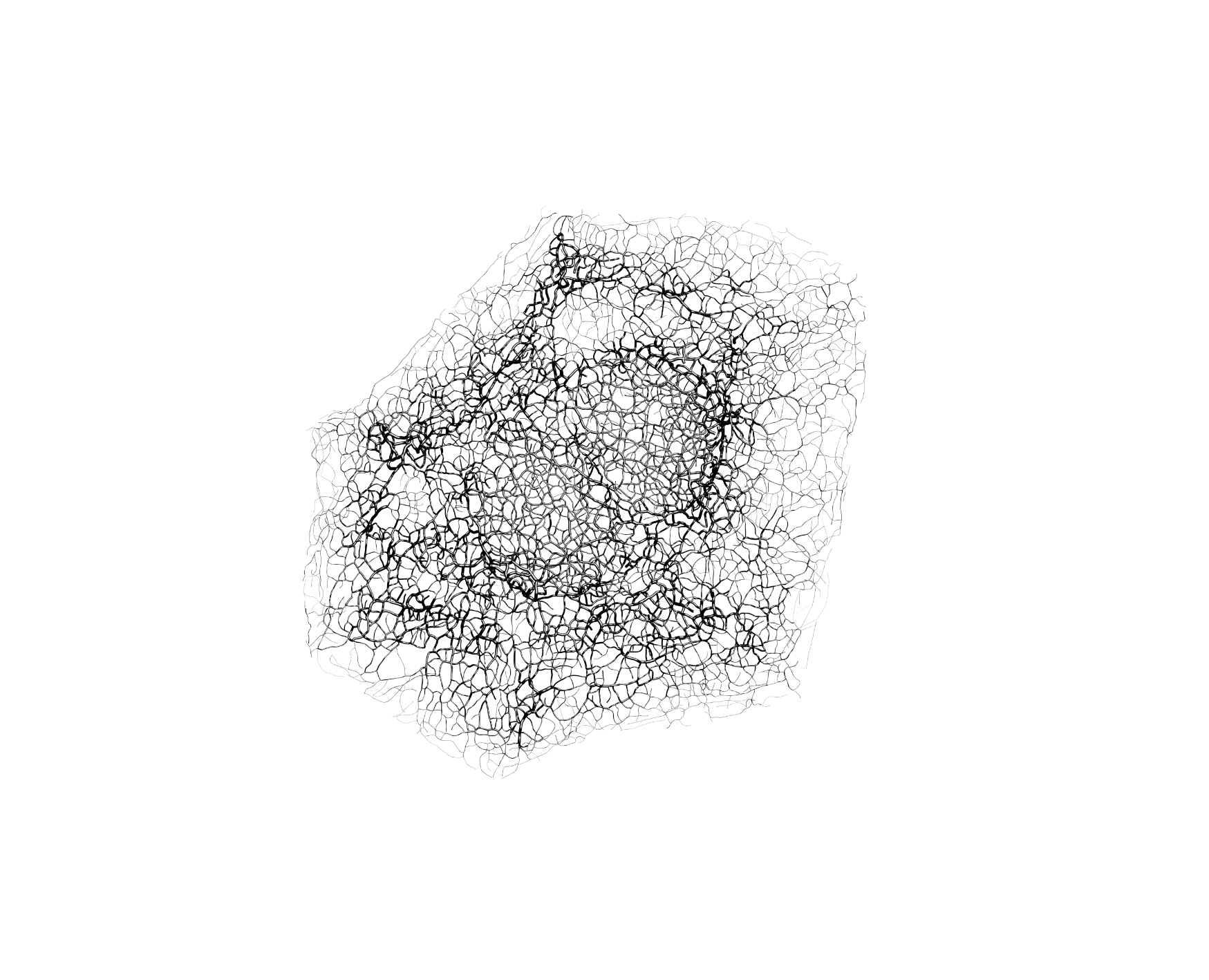

Supplement: Supplementary file 7 — Source data Fig. 5 [file 44319_2025_475_MOESM7_ESM.zip › Figure5/5F/Keratin rendering_Center.tiff]

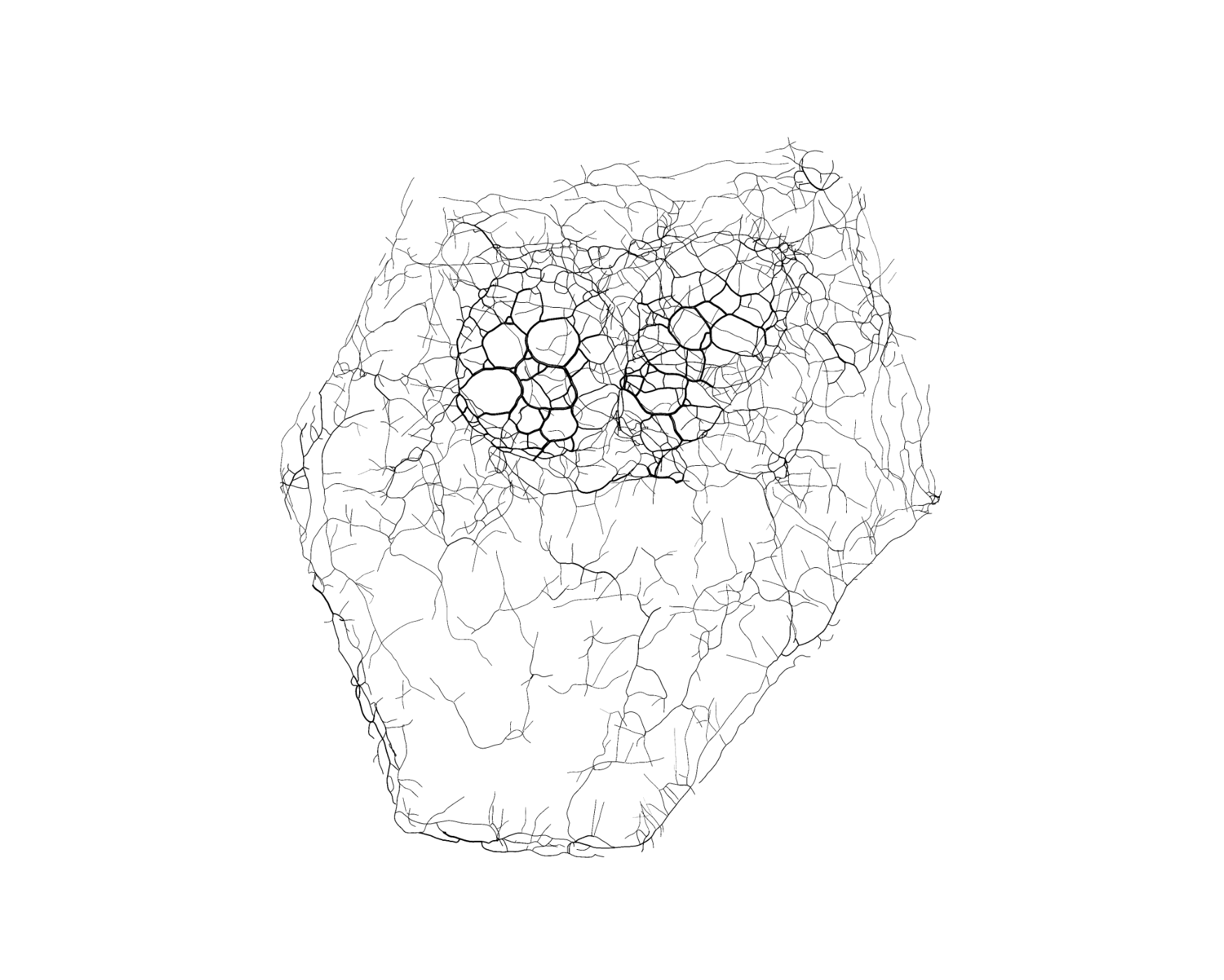

Supplement: Supplementary file 7 — Source data Fig. 5 [file 44319_2025_475_MOESM7_ESM.zip › Figure5/5F/Keratin rendering_FarPeriphery.tiff]

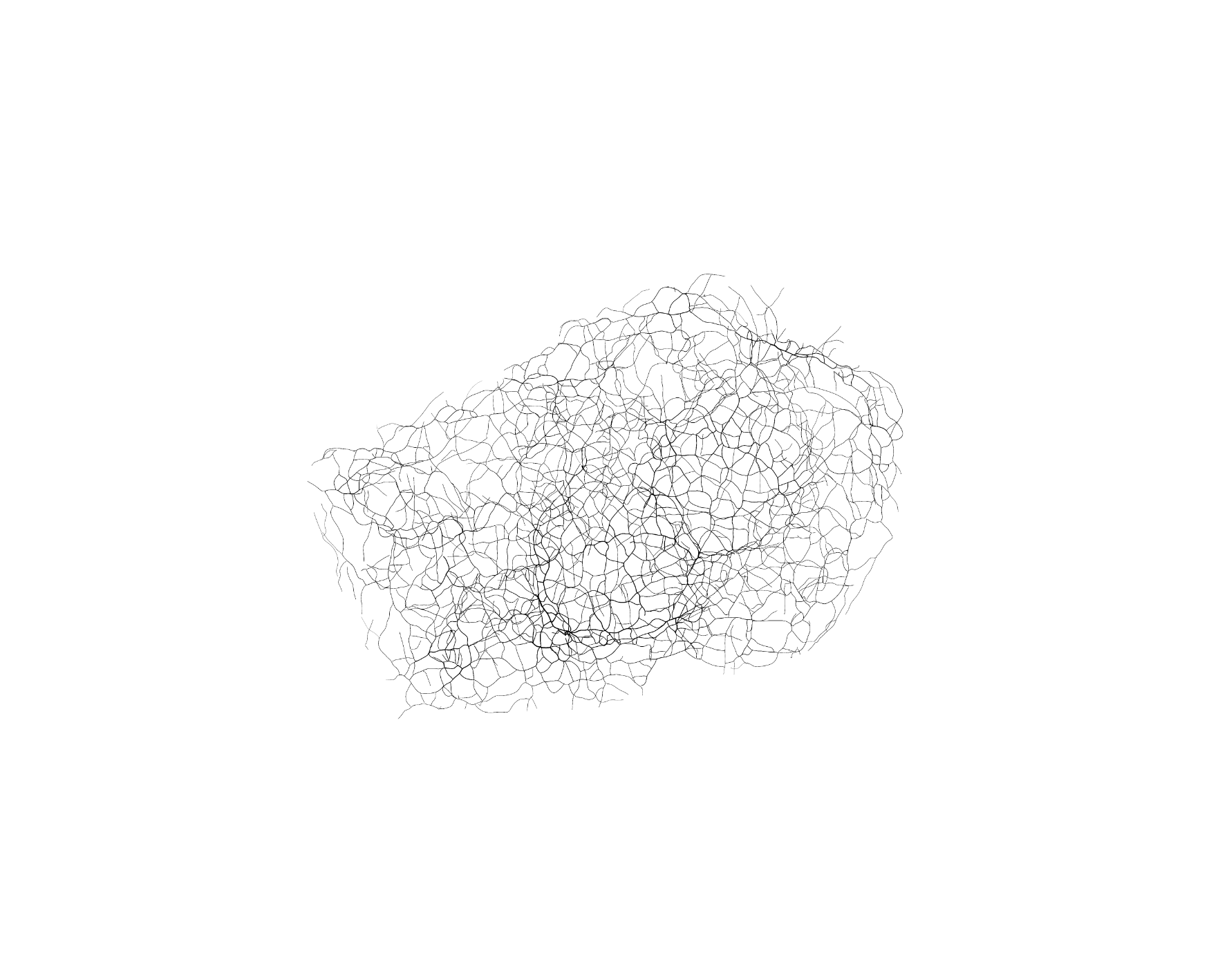

Supplement: Supplementary file 7 — Source data Fig. 5 [file 44319_2025_475_MOESM7_ESM.zip › Figure5/5F/Keratin rendering_MidPeriphery.tiff]

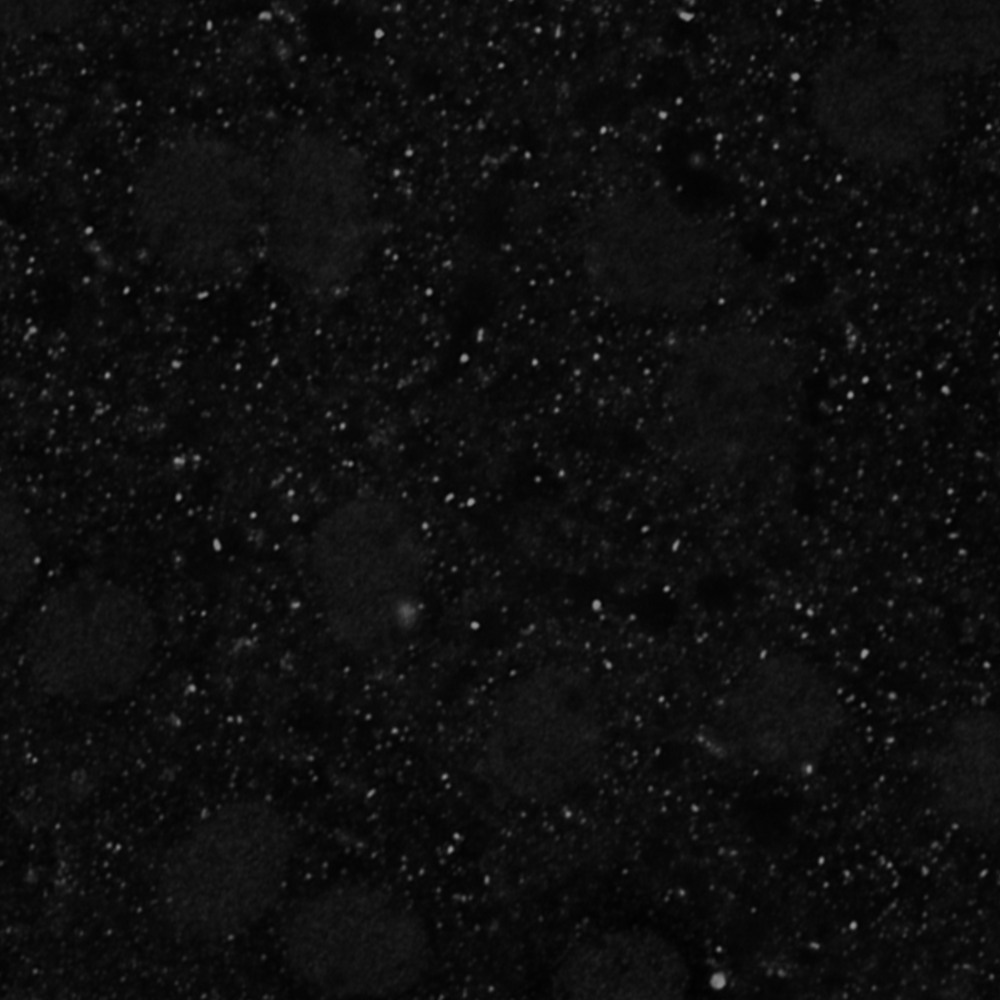

Supplement: Supplementary file 7 — Source data Fig. 5 [file 44319_2025_475_MOESM7_ESM.zip › Figure5/5C/Integrin b4_Centre.tif]

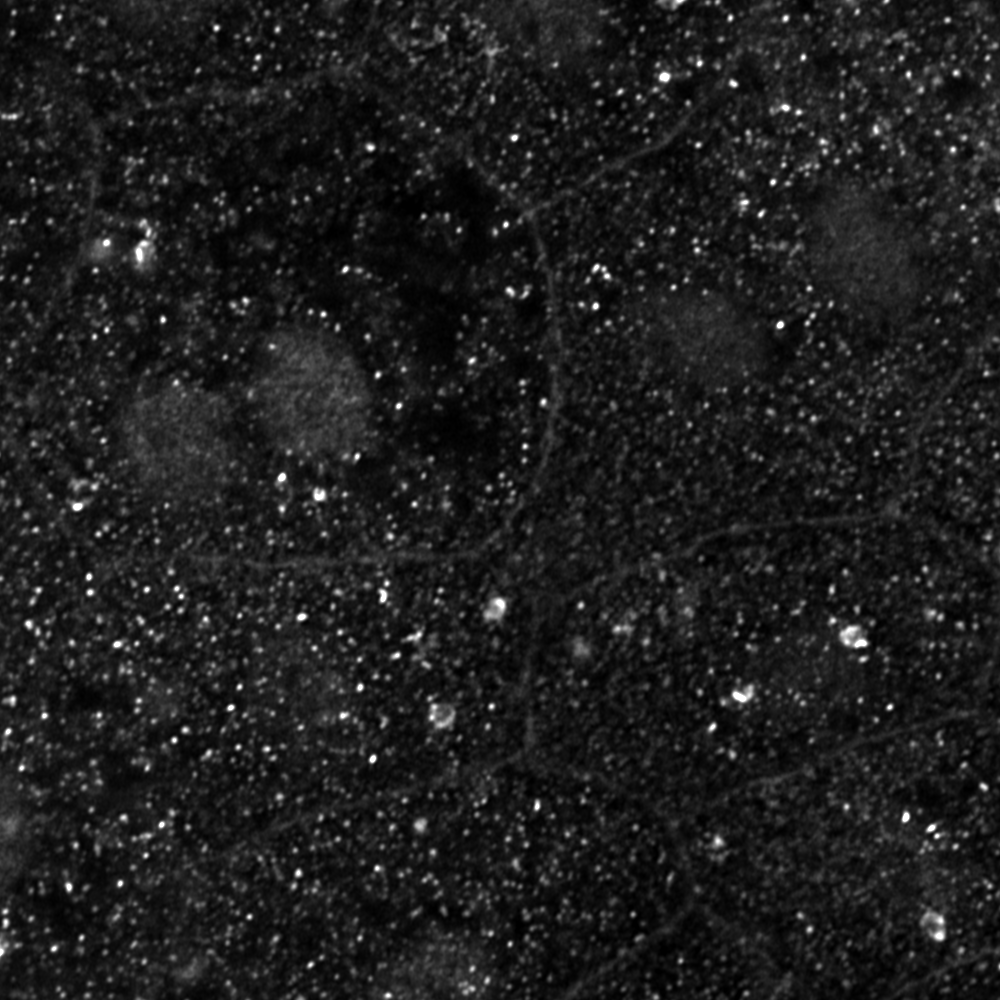

Supplement: Supplementary file 7 — Source data Fig. 5 [file 44319_2025_475_MOESM7_ESM.zip › Figure5/5C/Integrin b4_FarPeriphery.tif]

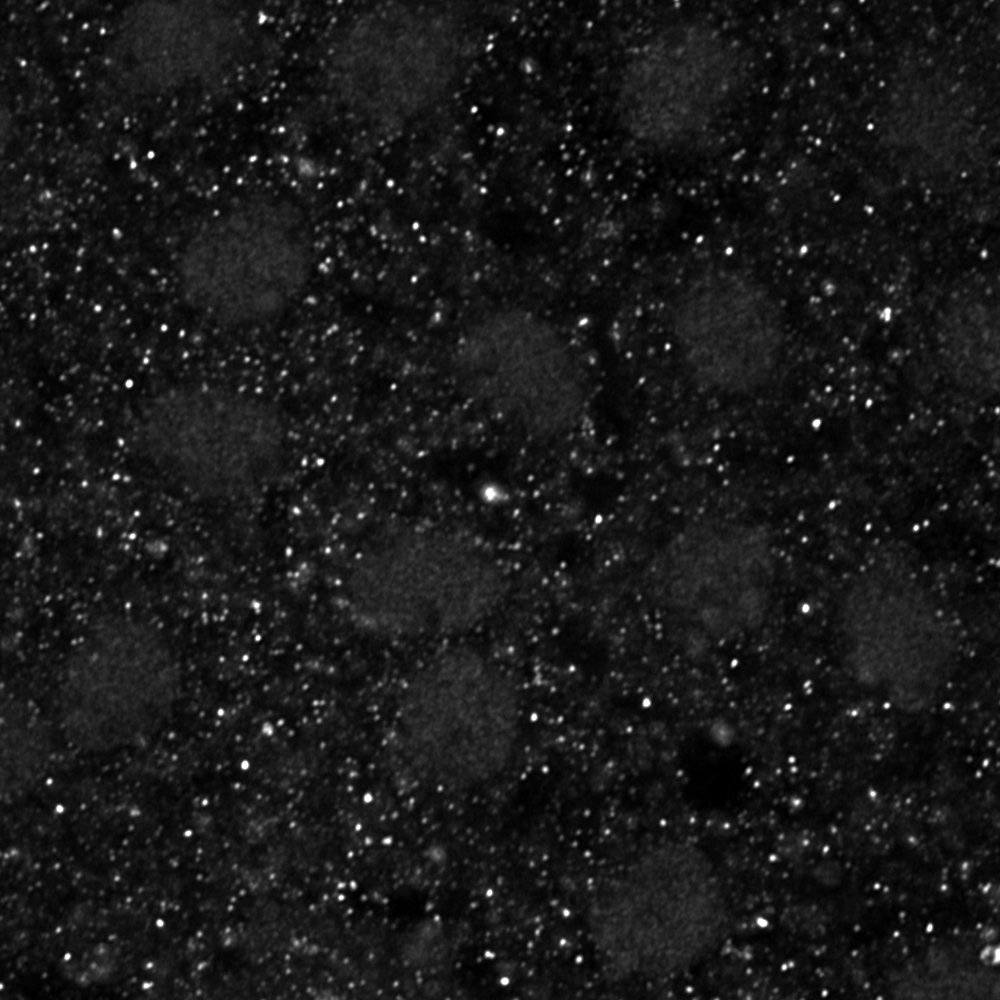

Supplement: Supplementary file 7 — Source data Fig. 5 [file 44319_2025_475_MOESM7_ESM.zip › Figure5/5C/Integrin b4_MidPeriphery.tif]

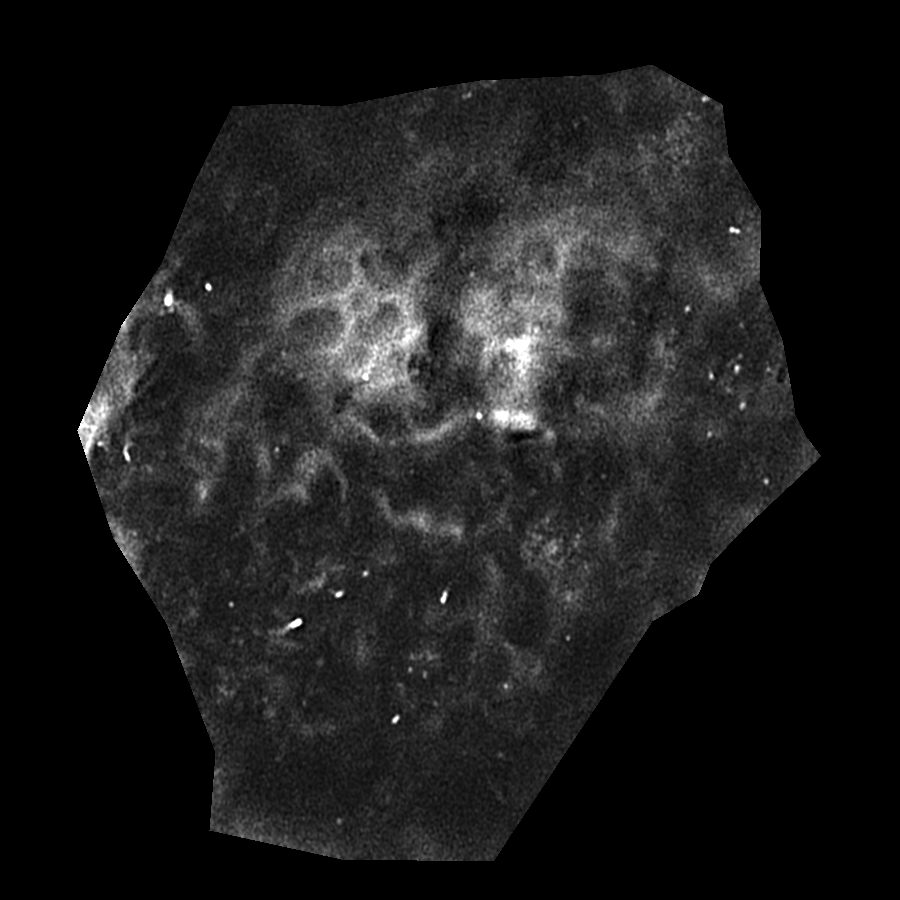

Supplement: Supplementary file 7 — Source data Fig. 5 [file 44319_2025_475_MOESM7_ESM.zip › Figure5/5E/Keratin_FarPeriphery.tif]

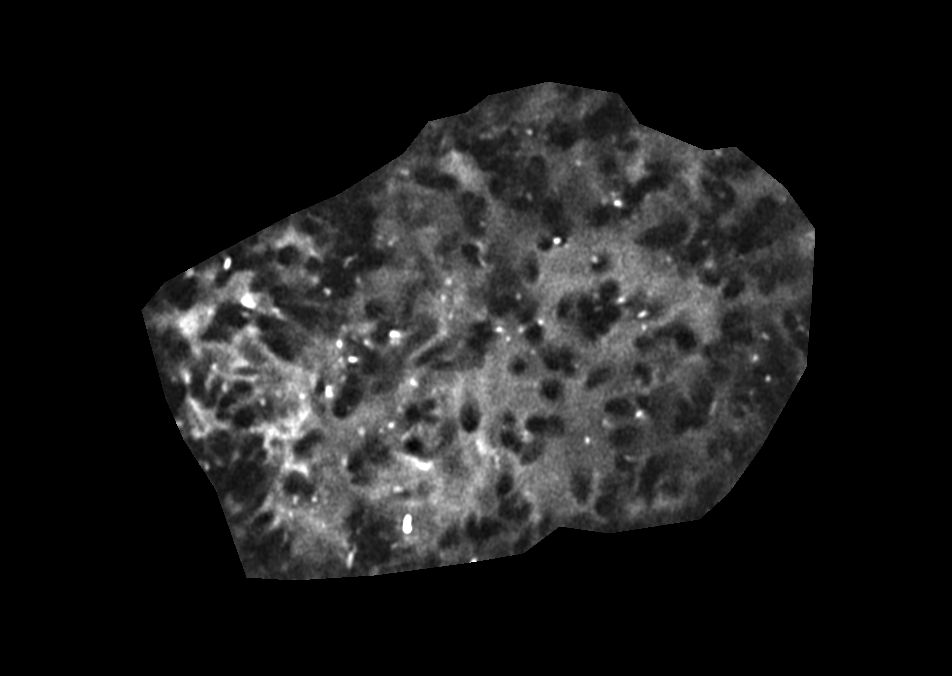

Supplement: Supplementary file 7 — Source data Fig. 5 [file 44319_2025_475_MOESM7_ESM.zip › Figure5/5E/Keratin_MidPeriphery.tif]

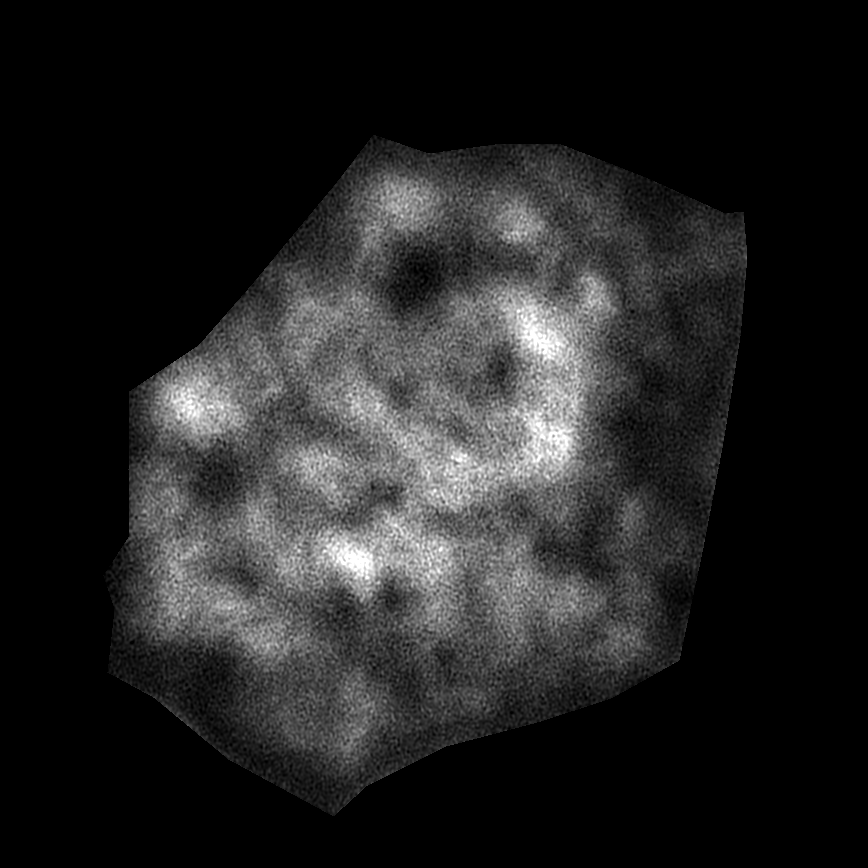

Supplement: Supplementary file 7 — Source data Fig. 5 [file 44319_2025_475_MOESM7_ESM.zip › Figure5/5E/Keratin_Centre.tif]
