# Supplementary material for: Laminin-defined mechanical status modulates retinal pigment epithelium phagocytosis
Source: EMBO Rep. 2025 May 19;26(13):3357–83. doi: 10.1038/s44319-025-00475-9 (PMC12238246; doi:10.1038/s44319-025-00475-9)
Supplement: Supplementary file 8 — Expanded View Figures [file 44319_2025_475_MOESM8_ESM.pdf]

Expanded View Figures

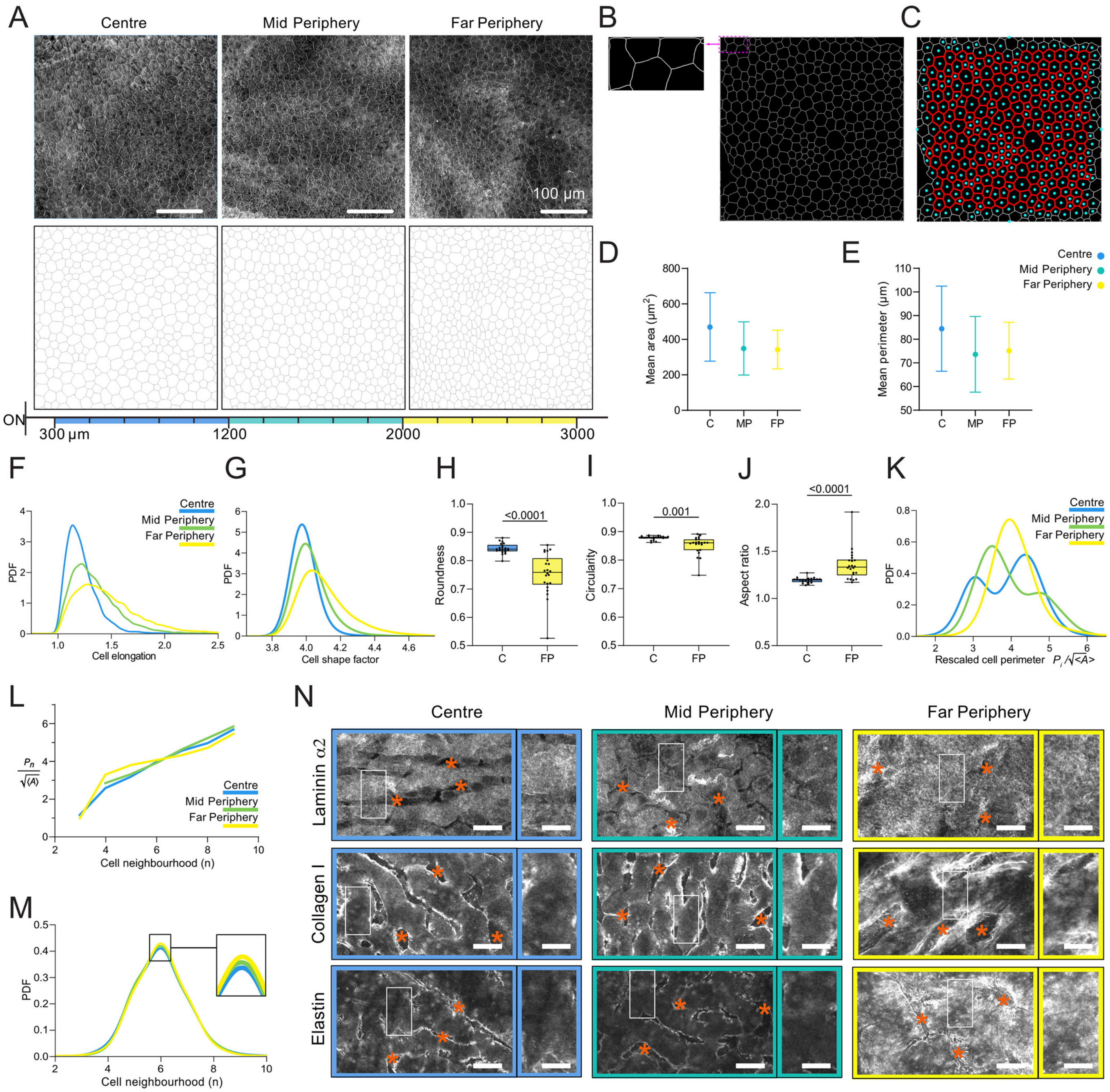

Figure EV1. Murine RPE represents spatially different monolayer organization.

(**A**) Representative confocal images of RPE stained for F-actin (top row) and post-processed binary images obtained by skeletonization used for the morphometric characterization upon segmentation (bottom row). Scale bar 100 μm. (**B**) Representative segmentation artefacts generated with Cellpose at the image edge. (**C**) The resulting cells (blue) and neighborhood (red) considered in the analyses. The average cellular area (**D**) and perimeter (**E**) shown with mean value and standard deviation for each region, respectively. Probability density function (PDF) of cell elongation (**F**) and cell shape factor (**G**) for different retinal regions. Nuclear geometric parameters such as roundness (**H**), circularity (**I**) and elliptical aspect ratio (**J**) show significant difference for cells from centre versus far periphery. The data was tested using Wilcoxon matched-pairs signed rank test; exact *p*-values are indicated on the figure for the respective comparisons. Box and whisker plots display the median (center line), 25th–75th percentiles (bounds of the box), and minimum to maximum values (whiskers). All individual data points are shown. (**K**) PDF of the rescaled cell perimeters defined as $P_i/\sqrt{<A>}$, Pi being the individual cell perimeter. Topological characteristics of the RPE monolayer such as Desh's law (**L**) representing the relationship between cellular perimeter and shape, and PDF of cell neighborhood (**M**) varying for three different regions. More than 5500 cells (technical replicates) were analyzed from 3 mice (biological replicates). (**N**) Representative immunofluorescent images of the Bruch's membrane stained for laminin α2, collagen type I and elastin coupled with magnified view of quantified regions. Stars indicate preparation artefacts. Scale bar 20 μm and 10 μm for main images and magnifications, respectively.

 © The Author(s)

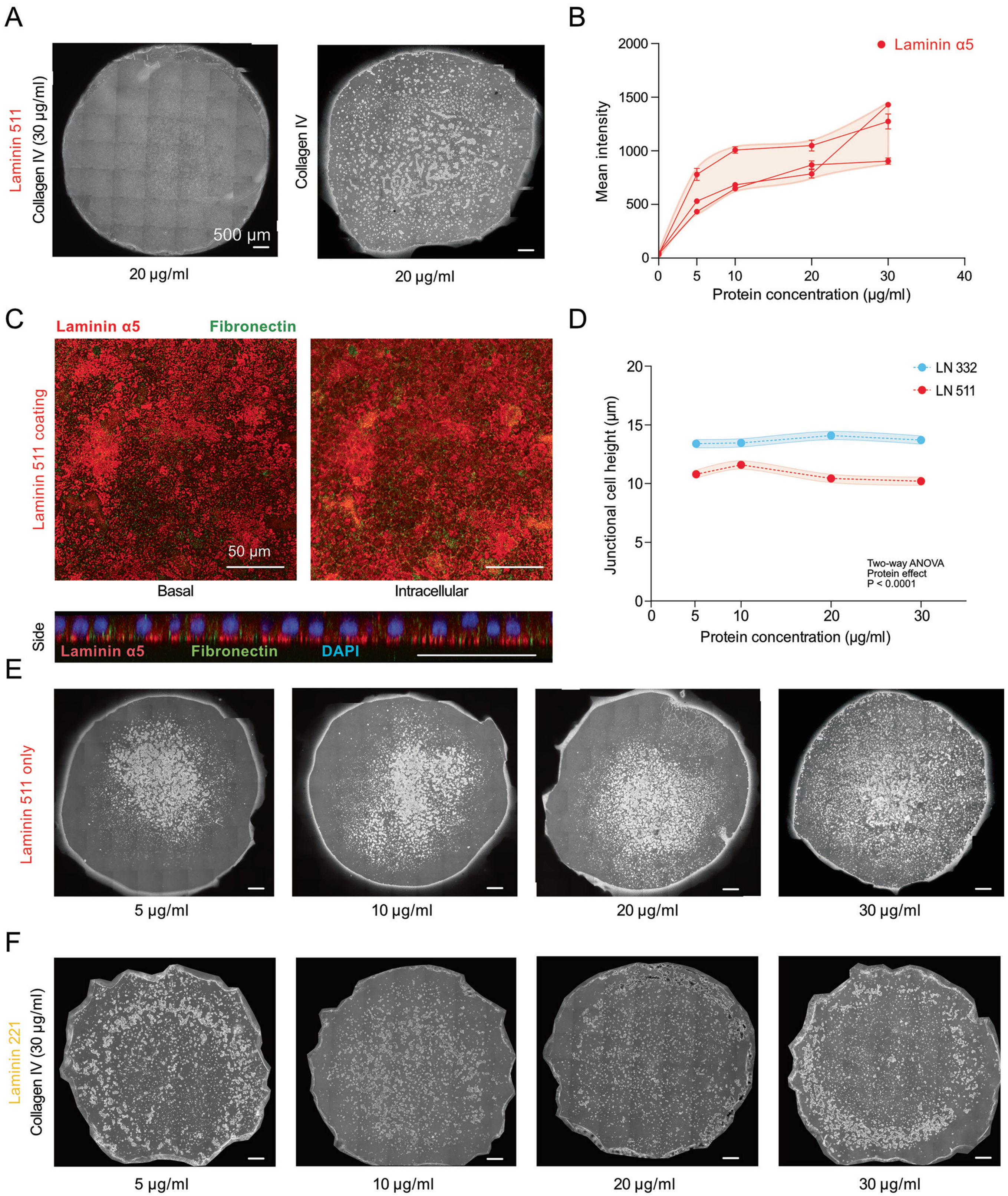
A
Laminin 511
Collagen IV (30 µg/ml)
500 µm
20 µg/ml
Collagen IV
20 µg/ml
B
Laminin α5
Mean intensity
Protein concentration (µg/ml)
C
Laminin α5
Fibronectin
Laminin 511 coating
50 µm
Basal
Intracellular
Side
Laminin α5
Fibronectin
DAPI
D
LN 332
LN 511
Junctional cell height (µm)
Two-way ANOVA
Protein effect
P < 0.0001
Protein concentration (µg/ml)
E
Laminin 511 only
5 µg/ml
10 µg/ml
20 µg/ml
30 µg/ml
F
Laminin 221
Collagen IV (30 µg/ml)
5 µg/ml
10 µg/ml
20 µg/ml
30 µg/ml

◀ **Figure EV2. Protein composition of polyacrylamide gel surface coating is crucial for cell adhesion strength.**

(**A**) Representative image of complete iRPE monolayer obtained on 4 kPa polyacrylamide gel coated with laminin 511 (20 μg/ml) combined with collagen type IV (30 μg/ml) in contrast with the fragmented monolayer on pure collagen type IV (20 μg/ml). Scale bar 500 μm. (**B**) Line graph of the mean fluorescent intensity of the hydrogel surface fixed and stained with laminin α5 antibody right after chemical crosslinking with different concentrations of laminin 511 combined with collagen type IV (30 μg/ml). This relation indicates correlation between laminin concentration and surface density. Each line represents individual dilution series. Images were taken from 10 different areas of the gel (technical replicates) from 3 independent experiments (biological replicates). Data are shown as mean within the gel ± SEM. (**C**) Immunofluorescent images of the laminin 511 coated gel surface stained for ECM proteins show the absence of fibronectin protein deposition after one week in cellular culture. Top row represents the signal from laminin α5 (red) and fibronectin (green) at the basal surface and within the monolayer. Fibronectin signal appears to be intracellular as shown in the side view of the monolayer. (**D**) Average cell height measured at the adherence junction levels of one-week-old iRPE monolayer. Measurements are the mean values from three fields of view (technical replicates) from at least three independent experiments (biological replicates) ± SEM. Statistic was performed using a mixed-effects model (REML) with the Geisser-Greenhouse correction, Tukey's multiple comparisons test. (**E**, **F**) Representative overview images of colonies iRPE cells seeded on gels coated with different concentrations of laminin 511 and laminin 221 combined with collagen type IV (30 μg/ml), indicating the insufficient cellular adhesion and monolayer formation in contrast to standard experimental conditions. Scale bar 500 μm.

 © The Author(s)

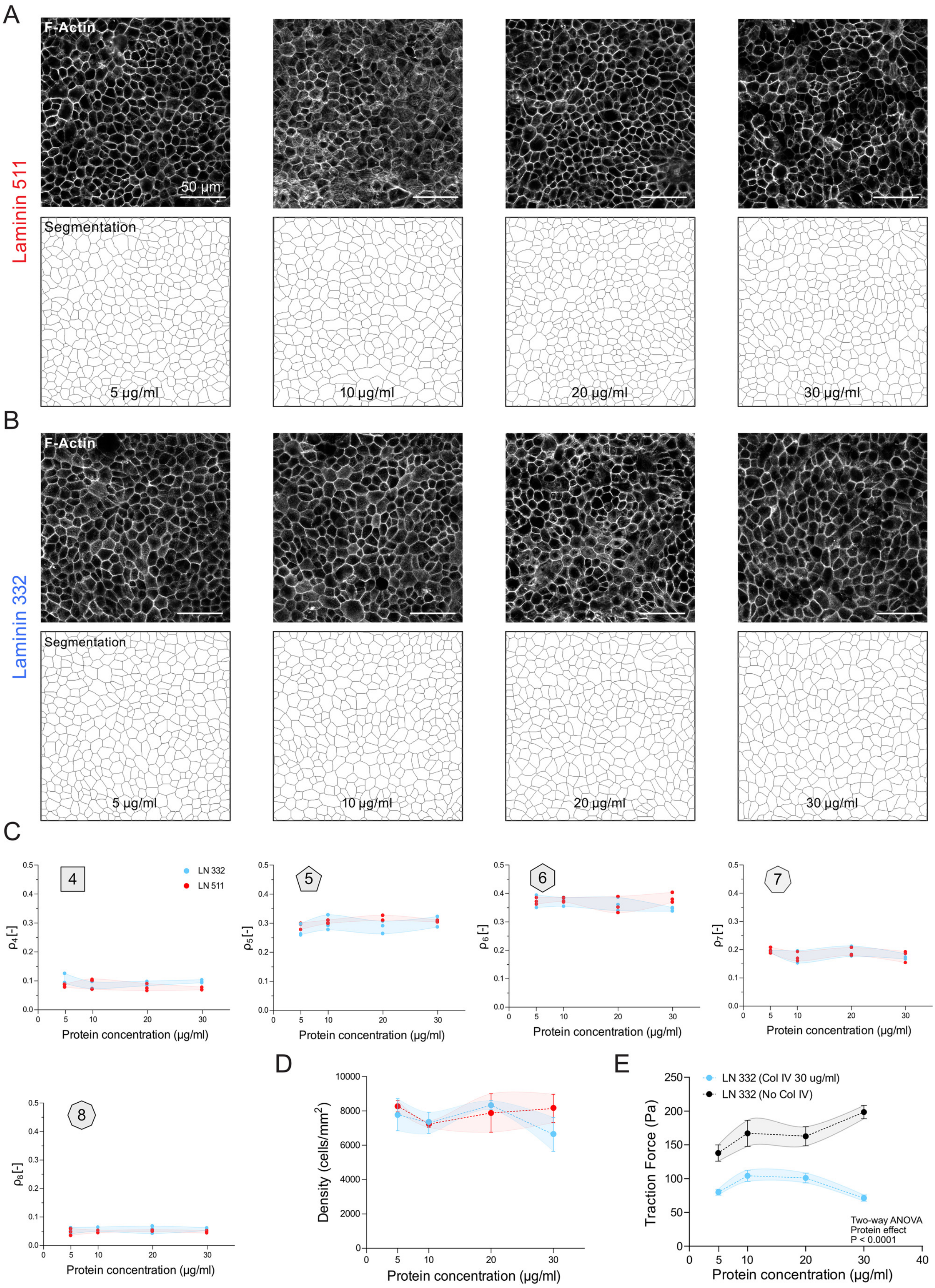
A
F-Actin
50 µm
Laminin 511
Segmentation
5 µg/ml
10 µg/ml
20 µg/ml
30 µg/ml
B
F-Actin
Laminin 332
Segmentation
5 µg/ml
10 µg/ml
20 µg/ml
30 µg/ml
C
LN 332
LN 511
ρ4 [-]
ρ5 [-]
ρ6 [-]
ρ7 [-]
ρ8 [-]
Protein concentration (µg/ml)
D
Density (cells/mm2)
Protein concentration (µg/ml)
E
LN 332 (Col IV 30 ug/ml)
LN 332 (No Col IV)
Traction Force (Pa)
Two-way ANOVA
Protein effect
P < 0.0001
Protein concentration (µg/ml)

Figure EV3. Structure and topological stability of iRPE in vitro.

Representative confocal images of one-week cultured iRPE monolayers on 4 kPa PAA gels coated with different concentrations of laminin 511 (**A**) or laminin 332 (**B**) both in presence of collagen type IV (30 µg/ml). The top row represents the actin cytoskeleton network in the stained samples and the bottom row shows segmented images used for morphometric analysis. (**C**) Probabilities of the cellular neighborhood (ρ_n) from 4 (ρ_4) “squares” to 8 (ρ_8) “octagon” indicating the stable neighborhood environment regardless of the protein surface concentration. Data are from 3 independent experiments (biological replicates) with 3 independent fields of view in each experiment (technical replicates). The total number of analyzed single cells is shown in Table EV1. (**D**) Quantification of cellular density across different experimental conditions shown as average between 3 biological replicates ± SEM. (**E**) The presence of collagen type IV in the laminin 332 containing coating solution decreases the traction forces exerted by cells. This difference becomes less significant at lower laminin concentration. Data are shown as mean of at least 4 biological replicates each with at least 6 technical replicates ± SEM. The statistical significance was assessed using mixed-effects model (REML) with the Geisser-Greenhouse correction and Tukey’s multiple comparisons test.

 © The Author(s)

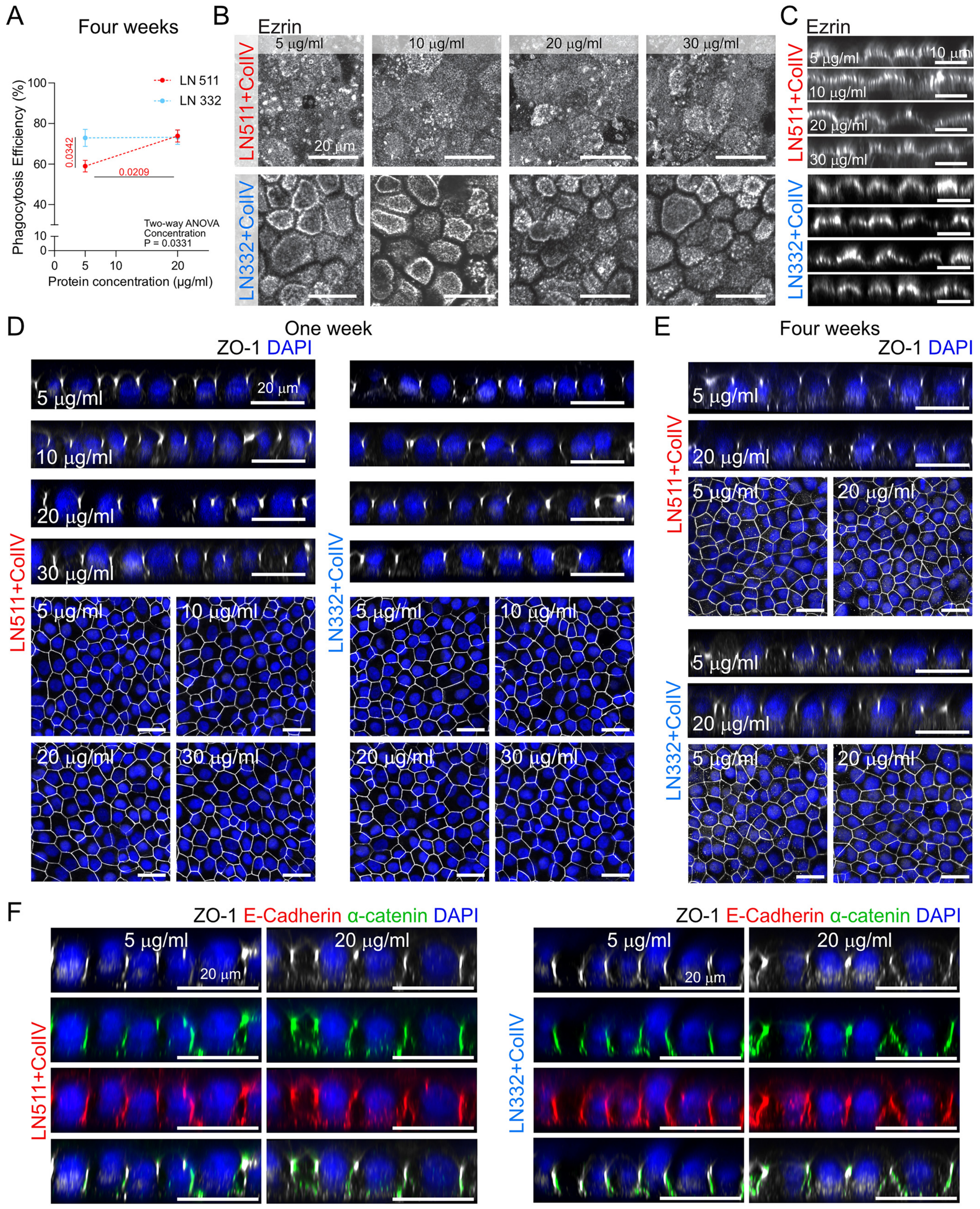
A
Four weeks
LN 511
LN 332
Phagocytosis Efficiency (%)
0.0342
0.0209
Two-way ANOVA
Concentration
P = 0.0331
Protein concentration (µg/ml)
B
Ezrin
5 µg/ml
10 µg/ml
20 µg/ml
30 µg/ml
20 µm
LN511+ColIV
LN332+ColIV
C
Ezrin
10 µm
D
One week
ZO-1 DAPI
E
Four weeks
ZO-1 DAPI
F
ZO-1 E-Cadherin α-catenin DAPI

◀ **Figure EV4. Effect of laminin coating on iRPE cellular maturity, polarization, and functionality.**

(**A**) Quantification of phagocytosis efficiency shown as an average between at least 3 technical replicates± SEM in 4-week-old iRPE cultures from 4 independent experiments (biological replicates), comparing different laminin isoforms and coating concentrations. The data were statistically tested using two-way ANOVA with Tukey's multiple comparison test. Exact *p* values are illustrated on the respective comparisons, with red values referring to multiple comparison test results. Representative Ezrin immunostaining overview (**B**) and orthogonal projections (**C**), representing microvilli organization across all laminin isoforms and concentrations. Orthogonal projections of tight junctions (ZO-1) and DAPI-stained nuclei, with XY plane images, for iRPE cultures cultured on different laminin isoforms and densities for one week (**D**) and four weeks (**E**). (**F**) Representative orthogonal projections showing localization of ZO-1, E-cadherin, alpha-catenin, and DAPI across both laminin isoforms at low and high coating densities.

© The Author(s)

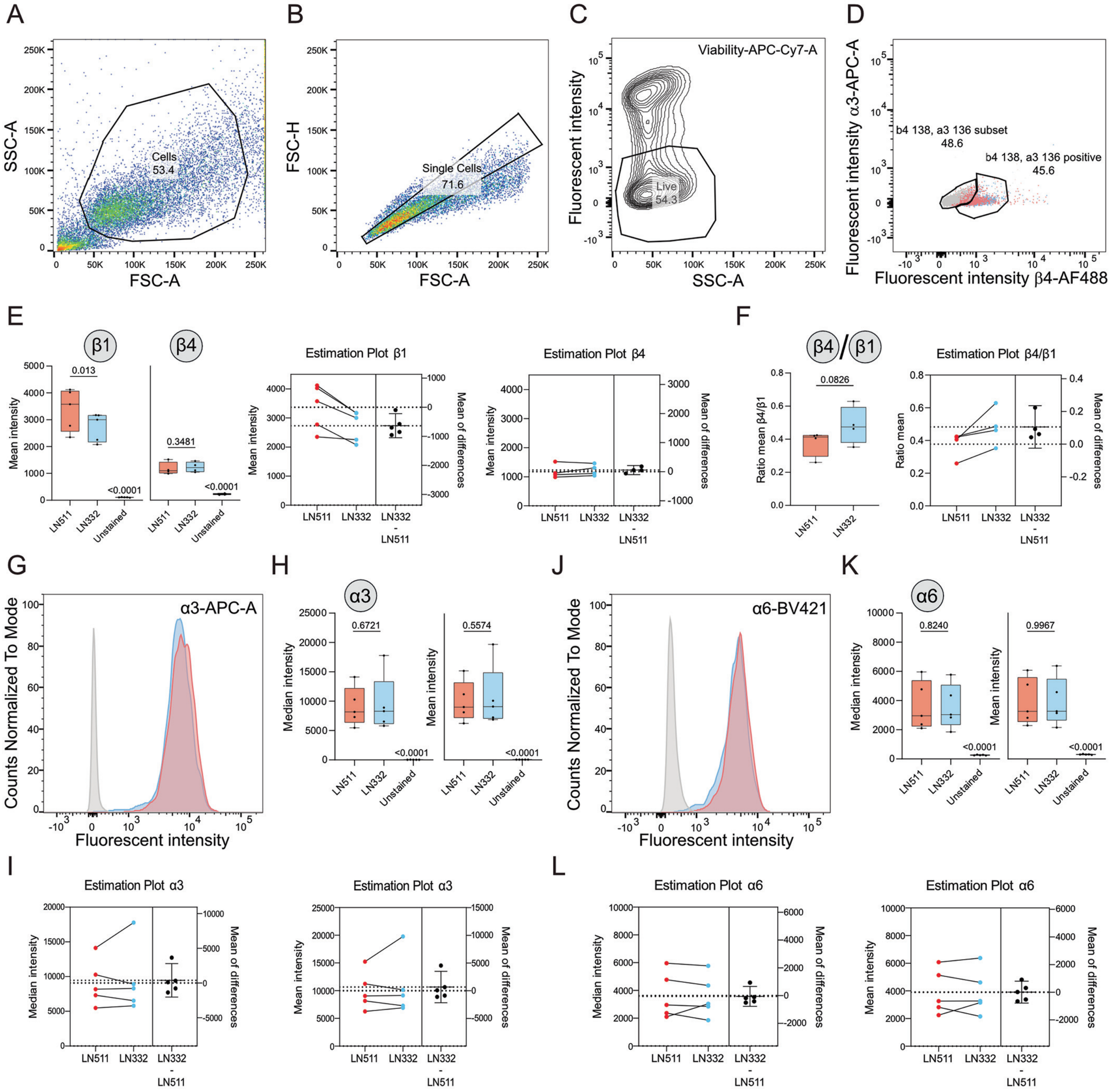

Figure EV5. Flow cytometry analysis of iRPE cultured on polyacrylamide gels coated with 5 μg/ml of laminin 511 or laminin 332.

Representative dot plots showing the gating strategy to identify cells (**A**), single cells (**B**), contour plot to extract a population of single living cells (**C**) and gating strategy for β4-positive cells (**D**). (**E**) Box and whisker plots of the mean intensity of integrin β1 and integrin β4 staining (left) and corresponding estimation plots (right) acquired from flow cytometry data. (**F**) Box and whisker (left) and estimation (right) plots showing the ratio between mean intensity of integrin β4 and integrin β1 staining. Representative histograms of flow cytometry analysis, box and whisker plots of median and mean fluorescent intensity and corresponding estimation plots of staining for APC-A-conjugated integrin α3 (**G–I**) and BV421-conjugated integrin α6 (**J–L**). The data was obtained from 5 independent experiments (biological replicates). Box and whisker plots display the median (center line), 25th–75th percentiles (bounds of the box), and minimum to maximum values (whiskers). All individual data points are shown. The gray-colored histogram represents the signal from unstained cells. Statistical analysis was done using a paired t-test to compare results between laminin 511 and laminin 332 coatings and one-way ANOVA with Dunnett's multiple comparison test for comparison with unstained sample. Exact *p* values are illustrated on the respective comparisons.
